# Human antibodies to SARS-CoV-2 with a recurring YYDRxG motif retain binding and neutralization to variants of concern including Omicron

**DOI:** 10.1038/s42003-022-03700-6

**Published:** 2022-07-29

**Authors:** Hejun Liu, Chengzi I. Kaku, Ge Song, Meng Yuan, Raiees Andrabi, Dennis R. Burton, Laura M. Walker, Ian A. Wilson

**Affiliations:** 1 Department of Integrative Structural and Computational Biology, The Scripps Research Institute, La Jolla, CA USA; 2 Adimab, LLC, Lebanon, NH USA; 3 Department of Immunology and Microbiology, The Scripps Research Institute, La Jolla, CA USA; 4 Ragon Institute of MGH, MIT and Harvard, Cambridge, MA USA; 5 Adagio Therapeutics, Inc, Waltham, MA USA; 6 The Skaggs Institute for Chemical Biology, The Scripps Research Institute, La Jolla, CA USA

**Keywords:** Viral infection, X-ray crystallography, SARS-CoV-2

## Abstract

Studying the antibody response to SARS-CoV-2 informs on how the human immune system can respond to antigenic variants as well as other SARS-related viruses. Here, we structurally identified a YYDRxG motif encoded by *IGHD3-22* in CDR H3 that facilitates antibody targeting to a functionally conserved epitope on the SARS-CoV-2 receptor binding domain. A computational search for a YYDRxG pattern in publicly available sequences uncovered 100 such antibodies, many of which can neutralize SARS-CoV-2 variants and SARS-CoV. Thus, the YYDRxG motif represents a common convergent solution for the human humoral immune system to target sarbecoviruses including the Omicron variant. These findings suggest an epitope-targeting strategy to identify potent and broadly neutralizing antibodies for design of pan-sarbecovirus vaccines and antibody therapeutics.

## Abstract (web summary)

Structural and computational analyses reveal a recurring YYDRxG motif in a class of human antibodies that target a conserved epitope on sarbecoviruses including the Omicron variant.

## Introduction

The current pandemic of coronavirus disease 2019 (COVID-19) has fomented devasting health, sociological and global economic consequences. Although several effective vaccines have been rapidly developed, SARS-CoV-2, the etiological cause of COVID-19, is still raging throughout the world. Vaccine efficacy has been affected by antigenic drift in SARS-CoV-2 that has led to enhanced infectivity as well as escape from neutralizing antibodies elicited by SARS-CoV-2 infection and vaccination. A majority of the potent neutralizing antibodies target the receptor binding domain (RBD) of the spike protein. However, these antibodies are often susceptible to mutations at the receptor binding site (RBS), which are frequently found in more challenging SARS-CoV-2 variants including Beta, Delta, and Omicron^1–9^. Notwithstanding, a subset of broadly neutralizing antibodies (bnAbs) can target highly conserved surfaces on the virus spike protein and neutralize circulating variants of concern (VOCs), variants of interest (VOIs), and other SARS-related viruses in the sarbecovirus family^9–19^. Identification and characterization of such cross-neutralizing antibodies are therefore urgently needed to fight the current COVID-19 pandemic, as well as to prepare for future potential zoonotic spillover.

Despite their broad spectrum of neutralization activity against SARS-CoV-2, circulating and emerging variants, as well as other related coronaviruses with high pandemic risk, potent cross-neutralizing antibodies are rarely isolated compared to specific SARS-CoV-2-neutralizing antibodies. These cross-neutralizing antibodies target regions in the spike proteins that are highly conserved across sarbecoviruses, which include the CR3022 site and N343 proteoglycan site in the RBD, as well as the S2 domain^2,10–18,20–27^. We and others have reported structures of cross-neutralizing antibodies targeting the CR3022 site that neutralize SARS-CoV-2, many circulating and emerging variants, and some other sarbecoviruses^11,13,16,27^. However, whether the human immune system is able to develop effective protection against all present and future SARS-CoV-2 variants and other sarbecoviruses has yet to be determined. Here, we identified a recurrent YYDRxG motif in ADI-62113 encoded by human *IGHD3-22* in heavy-chain complementarity-determining region 3 (CDR H3) from comparative analysis of the crystal structures of two cross-neutralizing antibodies, ADI-62113 and COVA1-16. A computational search of publicly available sequences with a YYDRxG sequence pattern led to identification of more such antibodies that are able to broadly neutralize SARS-CoV-2 VOCs including Omicron and SARS-CoV pseudoviruses, suggesting a general mechanism available to the human humoral immune system to combat SARS-related sarbecoviruses. Such information is critical for next-generation vaccine design and evaluation, as well as discovery of more effective therapeutic antibodies with increased breadth. Our study further suggests such cross-neutralizing bnAbs can potentially be rapidly identified from their sequence alone.

## Results

### Antibody ADI-62113 cross-reacts with a broad spectrum of sarbecoviruses

**Fig. 1:**
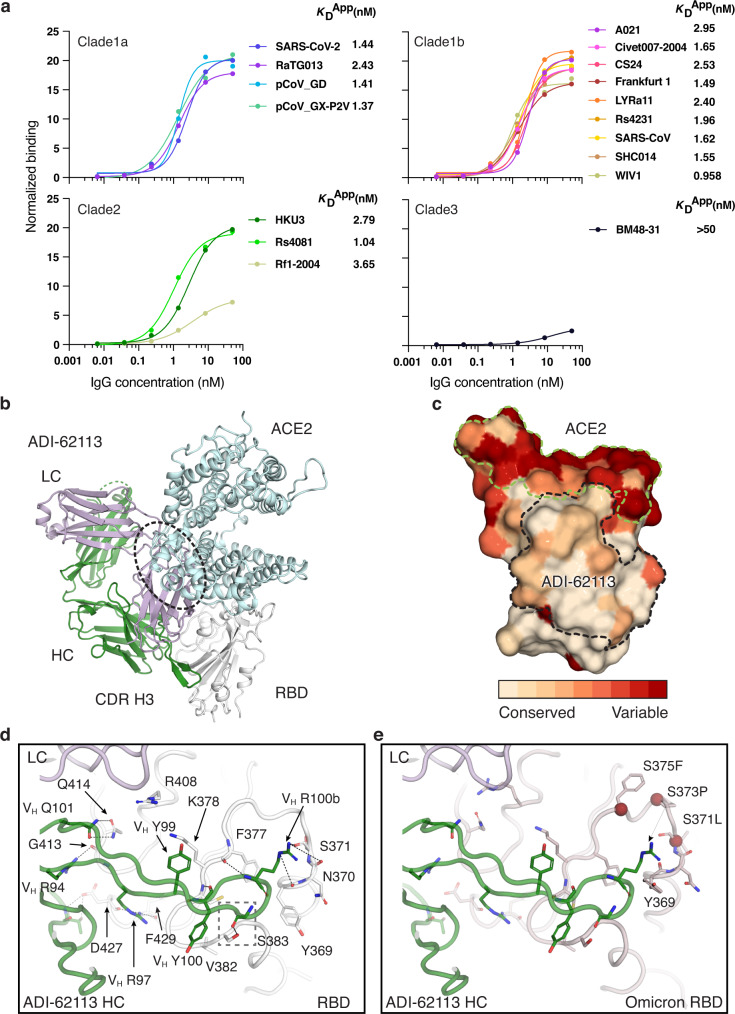
ADI-62113 binds a highly conserved site on SARS-CoV-2 RBD and cross-reacts with many sarbecoviruses. **a** ADI-62113 shows a broad spectrum of cross-reactivity to sarbecoviruses. RBDs from viruses in clade 1a (SARS-CoV-2-like viruses), clade1b (SARS-CoV-like viruses), clade 2, and clade 3 were displayed on the surface of yeast for binding kinetics analysis. Clade 1a and 1b viruses use ACE2 as an entry receptor. Clade 2 and 3 viruses do not bind ACE2 but contain homologous sequences to SARS-CoV-2. **b** Composite structure showing ADI-62113 competition with ACE2 binding for SARS-CoV-2 RBD. ADI-62113 Fab, ACE2, and SARS-CoV-2 RBD are shown in a ribbon representation. ACE2 is superimposed on the crystal structure of ADI-62113 with SARS-CoV-2 RBD based on PDB ID: 6M0J. White, RBD; pale cyan, ACE2; dark green, heavy chain; lavender, light chain. **c** ADI-62113 binds a highly conserved surface, while the receptor binding site (RBS) is highly variable across sarbecoviruses. SARS-CoV-2 RBD is shown in surface representation and colored by conservation across 169 sarbecovirus sequences (Supplementary Data 1). Dashed lines show the outline of the buried surface area (BSA) on the RBD by the human receptor ACE2 (green) and ADI-62113 (black). 81% of the ADI-62113 epitope surface is buried by the heavy chain and 19% by the light chain. CDR H3 contributes 69% of the total BSA. **d** CDR H3 interacts with highly conserved residues in the RBD. The crystal structure of ADI-62113 in complex with SARS-CoV-2 RBD is shown in tube representation. Residues involved in the interface between ADI-62113 and SARS-CoV-2 RBD are shown in sticks. Dashed lines represent hydrogen bonds or salt bridges. V_H_ R94, R97, R100b, G100d, and N101 hydrogen bond with the RBD. V_H_ Y99, Y100, and R100b have hydrophobic and amide-π interactions with the RBD. The dashed box indicates potential space constraints for the glycine in the YYDRxG motif in CDR H3 as shown in Fig. 2d. **e** The epitope site of ADI-62113 is much less impacted by mutations in VOCs such as Omicron BA.1. Epitope and paratope residues are shown in sticks with no labels (for labels, please see **d**). Mutation sites in Omicron are shown as red spheres with side chains as sticks and labeled with corresponding residue changes. A composite structure of ADI-62113+Omicron RBD was formed by superimposition of structures of ADI-62113+wildtype RBD (this study) and the Omicron RBD + ACE2 (PDB ID: 7T9L). The same perspective view as in **d** was used for parallel comparison. Interactions between ADI-62113 and RBDs are largely retained between wildtype (**d**) and Omicron RBD (**e**).

ADI-62113 is a potent cross-neutralizing antibody isolated from a COVID-19 patient^26^. Immunoglobulin heavy variable gene *IGHV1-3* and kappa variable gene *IGKV1-33* encode its heavy and light chain, respectively, and these germline genes have not been reported in other SARS-CoV-2 cross-neutralizing antibodies to date. To assess antibody breadth, we expressed various sarbecovirus RBDs on the surface of yeast to characterize their binding kinetics with antibody ADI-62113. ADI-62113 binds with high affinity to a broad spectrum of sarbecoviruses including ACE2-utilizing viruses in clade 1 and non-ACE2-utilizing viruses in clade 2 (Fig. 1a). Thus, its binding properties are highly favorable as a potential pan-sarbecovirus prophylactic or therapeutic.

### ADI-62113 binds a highly conserved site on SARS-CoV-2 RBD

To understand the basis of the broad reactivity of ADI-62113, we determined the crystal structure of ADI-62113 in complex with SARS-CoV-2 RBD at 2.6 Å resolution (Supplementary Fig. 1 and Table 1). ADI-62113 binds to the highly conserved CR3022 binding site^28^, but with an approach angle that can still block ACE2 receptor binding (Fig. 1b), which in part explains its high neutralization potency against SARS-CoV-2. The approach angle and binding mode is similar to another cross-neutralizing antibody, COVA1-16, which we reported previously^11^, although the heavy chain variable domain of COVA1-16 is encoded by a different germline gene *IGHV1-46*^11^ (Supplementary Fig. 1a).

**Table 1:** Crystallographic data collection and refinement statistics.

**Data collection**	
Beamline	SSRL 12-1
Wavelength (Å)	0.97934
Space group	*P* 4_3_ 2_1_ 2
Unit cell parameters	
a, b, c (Å)	85.0, 85.0, 215.0
α, β, γ (°)	90, 90, 90
Resolution (Å)^a^	50.0–2.59 (2.64–2.59)
Unique reflections^a^	25,315 (1,241)
Redundancy^a^	6.7 (5.8)
Completeness (%)^a^	99.2 (98.9)
<I/σ_I_>^a^	7.6 (1.1)
*R*_sym_^b^ (%)^a^	23.7 (>100)
*R*_pim_^b^ (%)^a^	9.7 (68.4)
CC_1/2_^c^ (%)^a^	97.9 (45.9)
**Refinement statistics**	
Resolution (Å)	45.4–2.59
Reflections (work)	23,894
Reflections (test)	1,170
*R*_cryst_^d^/*R*_free_^e^ (%)	22.1/26.7
No. of atoms	4,948
Macromolecules	4,838
Glycans	38
Solvent	72
Average *B-*value (Å^2^)	42
Macromolecules	42
Fab	41
RBD	45
Glycans	77
Solvent	40
Wilson *B*-value (Å^2^)	47
**RMSD from ideal geometry**	
Bond length (Å)	0.002
Bond angle (^o^)	0.50
**Ramachandran statistics (%)**^**f**^	
Favored	96.5
Outliers	0.0
**PDB code**	7T7B

^a^Numbers in parentheses refer to the highest resolution shell.

^b^*R*_sym_ = Σ_*hkl*_ Σ_*i*_ | I_*hkl,i*_ − <I_*hkl*_> | / Σ_*hkl*_ Σ_*i*_ I_*hkl,i*_ and R_*pim*_ = Σ_*hkl*_ (1/(n-1))^1/2^ Σ_*i*_ | I_*hkl,i*_ − <I_*hkl*_> | / Σ_*hkl*_ Σ_*i*_ I_*hkl,i*_, where I_*hkl,i*_ is the scaled intensity of the i^th^ measurement of reflection h, k, l, <I_*hkl*_> is the average intensity for that reflection, and *n* is the redundancy.

^c^CC_1/2_ = Pearson correlation coefficient between two random half datasets.

^*d*^*R*_cryst_ = Σ_*hkl*_ | *F*_o_ − *F*_c_ | / Σ_*hkl*_ | *F*_o_ | x 100, where *F*_o_ and *F*_c_ are the observed and calculated structure factors, respectively.

^e^*R*_free_ was calculated as for *R*_cryst_, but on a test set comprising 5% of the data excluded from refinement.

^f^From MolProbity^76^.

Further analysis of the antibody binding interface revealed that ADI-62113 CDR H3 dominates the interaction with SARS-CoV-2 RBD, contributing nearly 70% of the total buried surface area (BSA) on the RBD, as calculated by the PISA program^29^ (Fig. 1c). Its CDR H3 forms a β-strand like interaction with a SARS-CoV-2 RBD core β-strand through three main-chain to main-chain hydrogen bonds (Supplementary Fig. 1b). The aromatic ring of V_H_ Y99 interacts with aliphatic moiety of RBD K378 (Fig. 1d). V_H_ Y100 forms an amide-π interaction with the RBD peptide backbone of _382_VS_383_. V_H_ R94 interacts with the main-chain carbonyl of RBD G413, and V_H_ R97 with RBD D427 and F429 carbonyl oxygens. V_H_ Q101 forms equivalent hydrogen bond pairs with RBD Q414. The somatically mutated V_H_ R100b has multiple interactions with the RBD where its aliphatic moiety interacts with the aromatic ring of F377, and its guanidinium group forms three hydrogen bonds with the backbone of RBD _369_YNS_371_ and the partially negative dipole at the C-terminus of a short α-helix in the RBD, as also observed in COVA1-16^11^. This indented, negatively charged surface on the RBD is highly suited for engagement with V_H_ R100b (Supplementary Fig. 2a). Coincidentally, in the native spike trimer, this region is located in the interface between two adjacent RBDs, where this negative patch is engaged by R408 of a neighboring RBD in the “down” state, which may explain why arginine is naturally favored in ADI-62113 and COVA1-16 for interaction with this RBD site (Supplementary Fig. 2b). All of these RBD epitope residues are highly conserved across all 169 sarbecovirus sequences analyzed and are largely not changed in SARS-CoV-2 variants (Fig. 1c, d, Supplementary Fig. 3, and Supplementary Data 1), which supports the broad-spectrum cross-reactivity observed for ADI-62113 against sarbecoviruses (Fig. 1a).

### A YYDRxG motif shared between antibodies ADI-62113 and COVA1-16

**Fig. 2:**
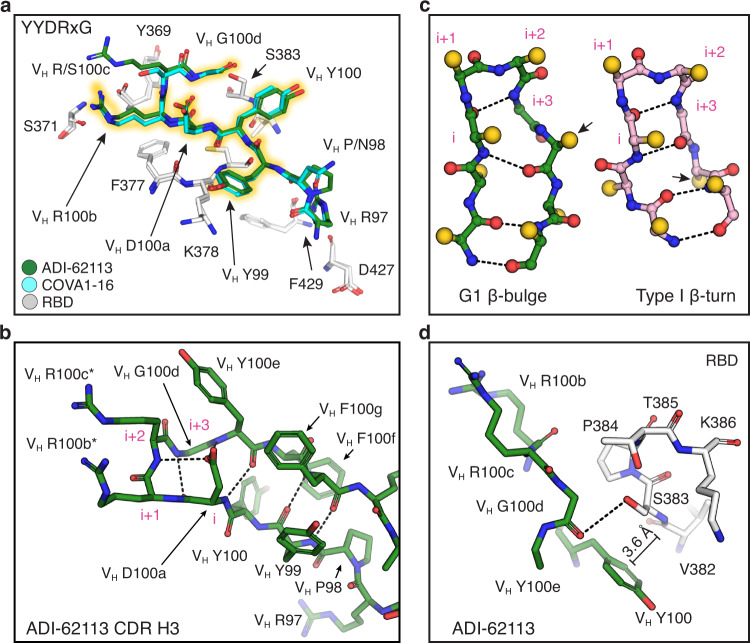
The YYDRxG motif is a recurring feature in CDR H3 for RBD binding. Hydrogen bonds are shown as dashed lines. Key epitope and paratope residues are shown in sticks and labeled. **a** Comparison of CDR H3 interactions of ADI-62113 and COVA1-16 with the RBD. White, SARS-CoV-2 RBD; green, ADI-62113; cyan, COVA1-16. The _99_YYDRxG_100d_ hexapeptide is highlighted in yellow to show its conserved structure in CDR H3 that interacts with the RBD. **b** The YYDRxG motif is located at the tip of CDR H3 and precedes a G1 β-bulge in the descending strand of the hairpin structure. Residues in the β-turn at the tip of CDR H3 are numbered i to i + 3 (magenta). The V_H_ D100a carboxyl (residue i) hydrogen bonds to backbone amide of V_H_ R100c (i + 2) in the β-turn as well as the backbone amide of V_H_ Y100e in the β-bulge. Asterisk (*) indicates somatically mutated residue. **c** Backbone comparison of an inserted β-bulge versus a standard β-strand in a β-sheet. Schematic backbones show the β-harpin in ADI-62113 CDR H3 that contains a G1 β-bulge following the glycine residue (i + 3) in the β-turn and comparison with a standard β-hairpin also with a type 1 β-turn at its tip (PDB ID: 4H5U). Amino-acid side chains are simplified as gold spheres. Arrows indicate the register change between the two motifs due to an additional residue in the β-hairpin in ADI-62113. **d** Limited space between residue V_H_ G100d and RBD favors a glycine residue at this position in the β-bulge. A hydrogen bond is formed between the V_H_ G100d carbonyl oxygen and S383 hydroxyl in the RBD. An amide-π interaction is formed between V_H_ Y100 and the peptide backbone of _382_VS_383_ of SARS-CoV-2 RBD.

In comparison to COVA1-16, we observed that CDR H3 of ADI-62113 exhibits near-identical interactions with the RBD despite differences in IGHV gene usage (Supplementary Fig. 1a, b). Interactions with other subsites seem have less impact on the binding mode as the different interactions of the ADI-62113 and COVA1-16 light chains with RBD R408 do not change the antibody approach angle or binding mode of CDR H3. Based on this comparative structural analysis, we could identify that the centerpiece of the antibody paratopes, the V_H 99_YYDRxG_100d_ hexapeptide, forms a conserved local structure for interaction with highly conserved residues in the RBD (Fig. 2a).

A β-bulge is formed near the tip of CDR H3 (Fig. 2b, c) after a type 1 β-turn (V_H 100a_DRRG_100d_ in this case) at its apex. An extra residue is inserted in the down strand of the β-hairpin at residue V_H_ Y100e and creates a G1 β-bulge, where the glycine residue in the bulge is in a left-handed α-helical conformation^30–32^. The carbonyl oxygen of V_H_ D100a forms a typical hydrogen bond in type I β-turns to the amide of V_H_ G100d. The negatively charged side-chain carboxyl of V_H_ D100a is then able to hydrogen bond to the backbone amides of V_H_ R100c in the β-turn and V_H_ Y100e in the β-bulge, which may further stabilize the local structure of the β-bulge.

Glycine residues are often found in a β-bulge and almost exclusively required in a specific position in a G1 β-bulge due to the specific dihedral constraints in the first position in traditional G1 numbering^30^ (n.b. the glycine is labeled here as i + 3 to show its position in the numbering of the preceding β-turn). V_H_ G100d has a positive phi (φ) angle (φ = 88°, ψ = 11°), consistent with a glycine residue in a G1 β-bulge, that alters the backbone conformation (Fig. 2c) and enables a hydrogen bond to be made between its carbonyl oxygen and the hydroxyl of the highly conserved RBD S383 (Fig. 2d). The presence of the additional residue due to the G1 β-bulge alters the subsequent register of the β-strand in the β-hairpin and allowing for accommodation of the additional residue^31^. The V_H_ Y100e side chain subsequently flips from a down configuration in the β-strand to an up configuration (Fig. 2c), thus opening up space for V_H_ Y100 to interact with the RBD (Fig. 2d). The insertion of an extra residue into the β-strand also accentuates the typical right-handed twist of the β-sheet^32^, which may contribute to the structural stability and specificity of CDR H3 in its interaction with the RBD (Supplementary Fig. 1). Moreover, the spatial constraint observed between the tip of CDR H3 of ADI-62113 and _382_VSPTL_386_ region in RBD may additionally drive the preference for a glycine in the β-bulge in ADI-62113 and COVA1-16 (Fig. 2d and Supplementary Fig. 2c). Overall, this structural comparison of two cross-neutralizing antibodies, ADI-62113 and COVA1-16, enabled identification of a unique pattern that includes the YYDRxG peptide where a register shift occurs in the β-hairpin through an insertion of a β-bulge that helps promote twisting of the β-hairpin, contributing to potential stabilization of this particular CDR H3 conformation for interaction with the RBD. Importantly, the YYDRxG motif contributes a combination of polar, hydrophobic, and amide-π interactions with the CR3022 site (Fig. 2a), providing a structural basis for the potent and broad neutralization against SARS-CoV-2, VOCs, and other SARS-related sarbecoviruses.

### YYDRxG pattern search identifies antibodies with D region encoded by *IGHD3-22*

**Fig. 3:**
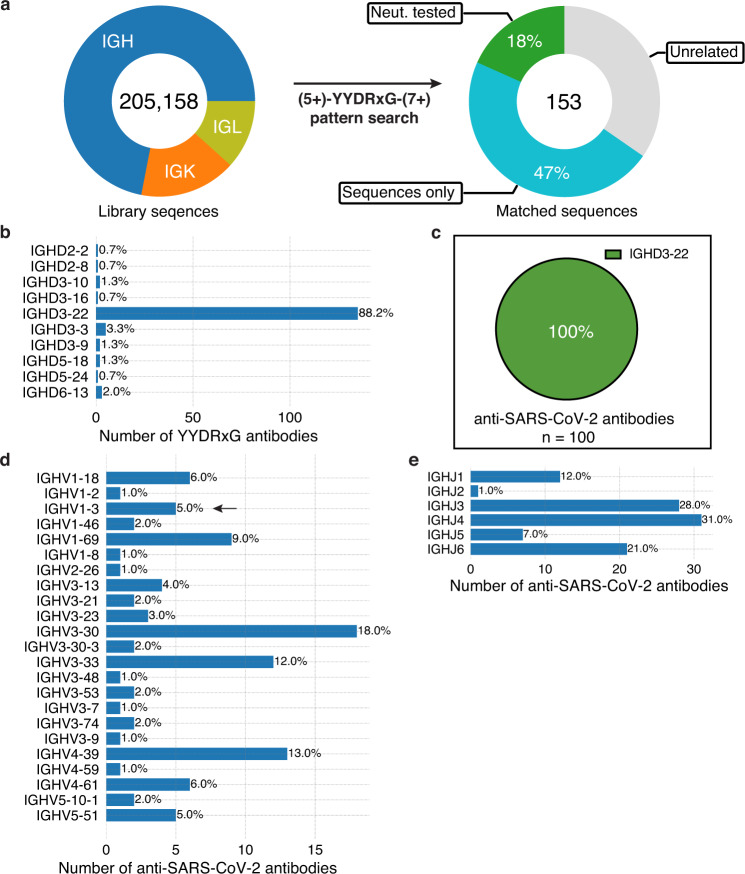
A YYDRxG pattern search identifies antibodies where the D region is encoded mostly by IGHD3-22. **a** Over 205,000 publicly available antibody sequences were retrieved from GenBank and supplementary files of previous publications reporting human anti-SARS-CoV-2 antibodies. The compiled antibody sequence library consists of 72% heavy chains (IGH), 16.4% kappa chains (IGK), and 11.7% lambda chains (IGL). CDR3 amino-acid sequences were used to search for the YYDRxG motif with five or more amino acid residues prior and seven or more amino acid posterior to the hexad motif. 153 heavy chain sequences were identified that contain a YYDRxG pattern and followed by data curation with literature review. 65% (100) are antibodies isolated from COVID-19 patients and mRNA vaccinees. 28 (18%) have been validated for SARS-CoV-2 neutralization (Neut. tested), while 72 (47%) have not (sequences only). **b** YYDRxG antibodies are mostly encoded by the *IGHD3-22* gene. All of the D genes used by YYDRxG antibodies were counted. Numbers at the right side of each bar indicate the percent of the total of identified YYDRxG antibodies. **c** *IGHD3-22* encodes all YYDRxG antibodies against SARS-CoV-2. All 100 sequences of anti-SARS-CoV-2 antibodies were analyzed and counted. **d**, **e** Heavy chain variable (**d** IGHV) and joining (**e** IGHJ) genes of YYDRxG antibodies isolated from COVID-19 patients or vaccinees. A diverse but limited set of variable and joining genes pair with *IGHD3-22* in YYDRxG antibodies against SARS-CoV-2. *IGHV3-30, IGHV4-39, IGHV3-33*, and *IGHV1-69* are more frequently found than others. Arrows indicates the immunoglobulin genes that encode ADI-62113 in this study.

Given these shared features were derived entirely from only two cross-neutralizing antibodies (ADI-62113 and COVA1-16), we sought to perform a computational pattern search to evaluate whether similar features might be present in other antibodies with sequences deposited in public databases (Fig. 3a). The CDR3 must be of sufficient length (≥18 residues by IMGT definition) to enable the YYDRxG hexapeptide to reach the conserved binding site on RBDs (Supplementary Fig. 2d). Hence, length constraints both N-terminal (≥5 aa) and C-terminal (≥7 aa) to the YYDRxG hexad were included in the YYDRxG pattern search. Homologous sequence to the YYDRxG hexapeptide were also used in the search since V_H_ Y99, V_H_ Y100, and V_H_ R100b all form hydrophobic interactions with the RBD, which may be possible for other residues containing hydrophobic moieties. In a search of over 205,000 antibody sequences, 153 antibodies with an YYDRxG pattern in their CDR H3 were identified (Supplementary Tables 1–2 and Supplementary Fig. 4a). Immunoglobulin gene analysis shows that the *IGHD3-22* gene is highly enriched in 88% of these antibodies (Fig. 3b), which is the same diversity (D) gene used in ADI-62113 and COVA1-16 (Supplementary Fig. 1a), compared to 8.5% in the overall search library (Supplementary Fig. 4b).

Of these 153 antibodies, 100 (65%) were isolated from cohorts consisting of COVID-19 patients and mRNA vaccinees^26,33–43^. All of the D regions are exclusively encoded by *IGHD3-22* (Fig. 3c and Supplementary Tables 1 and 2). In contrast, there does not appear to be any strict constraint on IGHV and IGHJ gene usage (Fig. 3d, e), consistent with the different IGHV gene usage of ADI-62113 and COVA1-16. Diverse sets of IGHV and IGHJ genes are paired with *IGHD3-22*, which are distinct from, but overlap with, YYDRxG antibodies identified in non-COVID cohorts (unrelated sources) (Supplementary Fig. 4c, d). Some IGHV genes are more often paired with *IGHD3-22* such as *IGHV3-30, IGHV4-39, IGHV3-33*, and *IGHV1-69* (Fig. 3d), although the current number of antibodies available for analysis (*n* = 100 for anti-SARS-CoV-2 antibodies vs. *n* = 53 for other antibodies) limits conclusive determination of whether IGHV gene pairing preferences exist for this particular subset of SARS-CoV-2 antibodies.

### Somatic hypermutations, specific reading frame, and N additions to *IGHD3-22* are required

**Fig. 4:**
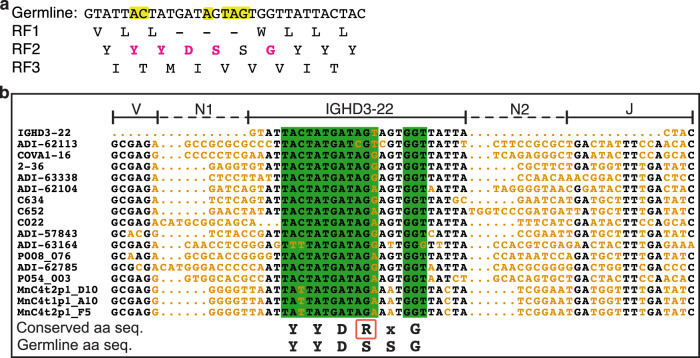
Reading frames (RFs) and sequence alignment of *IGHD3-22*. **a** Three possible RFs of the *IGHD3-22* germline sequence were translated into amino-acid sequences. Only RF2 encodes the YYDRxG motif (magenta). Nucleotides highlighted in yellow in the germline DNA sequence indicate sites where somatic hypermutations are often found in YYDRxG neutralizing antibodies against SARS-CoV-2. – indicates a stop codon. **b** Alignment of YYDRxG coding sequences in CDR H3. Conserved nucleotide sequences are in black, and variable sequences in orange. N additions (N1 and N2) as defined by IMGT junction analysis^75^ are indicated by dashed lines. *IGHD3-22*, IGHV(V), and IGHJ (J) regions are indicated by solid lines above the sequences. Sequences in a green background encode the YYDRxG key residues. Conserved YYDRxG sequence and corresponding germline amino-acid sequence (aa seq.) are shown below the sequence alignment panel for comparison. The arginine residue in the YYDRxG motif in a red box resulted from a high co-incidence of somatic mutations. Only the first 16 coding sequences are shown in the sequence alignment to represent 100 such antibodies against SARS-CoV-2.

The antibody coding sequence is a product of immunoglobulin gene rearrangement, namely V(D)J recombination, non-templated addition (N addition), and somatic hypermutation during B cell development. The V(D)J junctional sequences encode CDR H3. Three reading frames (RFs) are possible for *IGHD3-22* due to insertion of N additions between the VH and D regions. Here the second RF is exclusively observed in the YYDRxG antibodies since the other two result either in early termination of translation (RF1) or a very different sequence (RF3) (Fig. 4a). The situation differs from the *IGHD3-9* gene that is commonly used in broadly neutralizing anti-influenza stem antibodies, where two of the three RFs are used to encode CDR H3^44^. Moreover, alignment of CDR H3 coding sequences of representative neutralizing antibodies against SARS-CoV-2 shows a high incidence of T → A/G or A → C transversions converting serine in the germline sequence to arginine (Fig. 4b), supporting the hypothesis that somatic mutation from the germline serine to arginine residue in the YYDRxG motif (V_H_ R100b in ADI-62113 or COVA1-16) is critical for high affinity binding and neutralization^11^. We also observe frequent somatic mutations adjacent to the serine codon (Fig. 4a, b), which may be a lesion site created during antibody affinity maturation and serve as a prerequisite of converting S100b to R100b in the germinal center as somatic hypermutation of A:T pairs requires additional mutagenic processes at neighboring sites^45,46^. In addition to the S100b to R100b mutation in ADI-62113, mutation in a neighboring codon then leads to a somatically mutated R100c (Figs. 2b and 4b). While V_H_ R100b is critical for binding the RBD, V_H_ R100c has no interaction with RBD as indicated by the paucity of electron density for the V_H_ R100c side chain (Supplementary Fig. 2c). Thus, V_H_ R100c is not absolutely required since a serine at the same position does not impact binding, as observed in COVA1-16 (Supplementary Fig. 1b). N additions (N1 and N2) at both ends of *IGHD3-22* during V(D)J recombination are also important in determining both CDR H3 length and the reading frame of *IGHD3-22* that are critical for positioning and interaction of YYDRxG with the RBD. Overall, the requirement for a specific RF, site-specific somatic hypermutation, and relatively long N additions at both ends of *IGHD3-22* may contribute to the comparatively low frequency of YYDRxG motif in the human antibody repertoire, which may in part explain the relatively rare occurrence of such cross-neutralizing antibodies isolated to date from COVID-19 patients and vaccinees.

### YYDRxG antibodies are associated with broad neutralization against all VOCs including Omicron

**Fig. 5:**
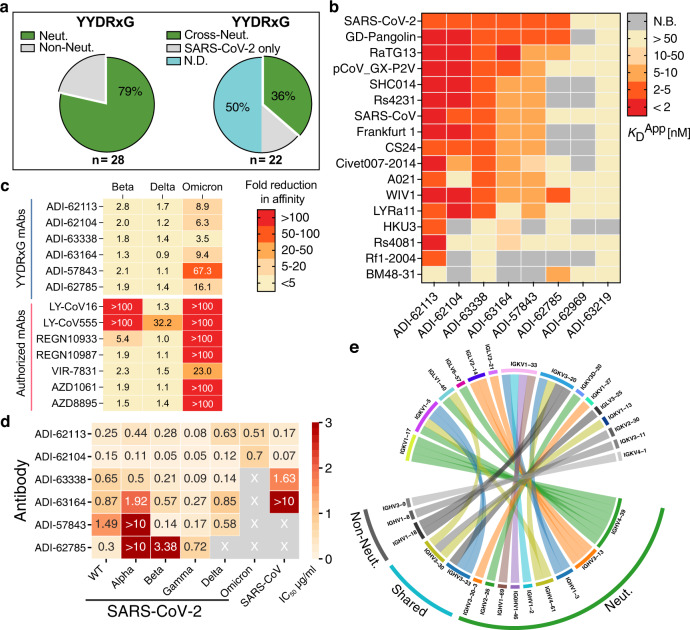
YYDRxG antibodies are associated with broad neutralization against VOCs including Omicron. **a** Neutralization overview of YYDRxG antibodies against SARS-CoV-2. Antibodies with neutralization data available from this study or from previous publications are included in this analysis. Within 28 YYDRxG antibodies, 22 (79%) exhibit neutralization (Neut.) against SARS-CoV-2 (left). Among these 22 antibodies, 8 (36%) cross-neutralize (Cross-Neut.) SARS-CoV, while 3 (14%) do not (SARS-CoV-2 only); another 11 (50%) have not been tested against SARS-CoV (N.D.) (right). **b** Cross-reactivity of ADI antibodies with a YYDRxG motif across sarbecoviruses. ADI antibodies were titrated with sarbecovirus RBDs expressed on the yeast surface. Color bars indicate apparent binding affinity (*K*_D_^App^) as in the key. Red indicates strong binding, yellow indicates weak binding, and gray indicates no detectable binding (N.B.). Most antibodies are cross-reactive with many other sarbecovirus RBDs, except for ADI-63219 and ADI-62969. ADI-62113 showed the broadest spectrum of cross-reactivity to other sarbecoviruses. Titration curves used to determine binding affinities are shown in Supplementary Fig. 5. **c** YYDRxG antibodies generally preserve their binding to more challenging variants with strong escape mutations or increased transmissibility, such as Beta, Delta, and Omicron, while almost all antibodies under emergency use authorization do not. Kinetics for each antibody in binding to RBDs of the indicated SARS-CoV-2 variants were measured and compared to binding to the RBD of the wildtype ancestral virus. Fold reduction in affinity (*K*_D_) was plotted with red indicating substantial loss of binding (>100-fold reduction) and yellow indicating no significant change (<5-fold reduction). Titration curves used to determine binding affinities are shown in Supplementary Fig. 6. **d** ADI antibodies neutralize SARS-CoV-2, VOCs and SARS-CoV. Neutralization was tested using a pseudovirus assay system. Neutralization potency, i.e., IC_50_, for each antibody against corresponding viruses are shown on a heatmap with wheat indicating potent neutralization. X indicates weak to no neutralization activity. ADI-63219 and ADI-62969 showed no neutralization against SARS-CoV-2 and are not included in this heatmap. Titration curves used to determine neutralization potency are shown in Supplementary Fig. 7. **e** Distinct combinations of heavy and light variable genes used by neutralizing and non-neutralizing antibodies. Circos plot showing combinations of heavy and light chain variable genes in each encoded antibody. Colored ribbons represent variable genes encoding neutralizing antibodies (Neut.) while gray indicate non-neutralizing antibodies (Non-Neut.). *IGHV3-30, IGHV3-33*, and *IGKV3-20* were found in both neutralizing and non-neutralizing antibodies. YYDRxG antibodies against SARS-CoV-2 with neutralization data available are included in this analysis (*n* = 28). Antibody names and CDR H3 sequences of these antibodies are included in Supplementary Table 1.

Among antibodies identified with a YYDRxG motif, 28 (18%) have been experimentally characterized via SARS-CoV-2 neutralization assays (Fig. 3a, right panel). 25 of these 28 (89%) antibodies recognize SARS-CoV-2 RBD and 22 (79%) have been reported to effectively neutralize the virus in previous publications (Fig. 5a and Supplementary Table 1)^16,26,33,36–39,43^. However, data for cross-reactivity to VOCs and other sarbecoviruses are incomplete. To determine whether the YYDRxG motif is associated with broad antigen recognition, we tested eight available ADI antibodies^26^ against a panel of sarbecovirus RBDs. We observed that most of these antibodies, except for ADI-63219 and ADI-62969, strongly cross-react with many representative sarbecoviruses with apparent disassociation constants (*K*_D_^App^) ranging from 1.0 to 30.6 nM (Fig. 5b and Supplementary Fig. 5). Despite the presence of the YYDRxG hexapeptide, ADI-62969 and ADI-63219, show weak affinity to all sarbecovirus RBDs tested including SARS-CoV-2. However, ADI-62969 and ADI-63219 have shorter or longer CDR H3 (19 or 25 residues by IMGT definition) compared to the others (average of 22 with a total range of 19-26 residues) that may restrict the ideal positioning of YYDRxG for RBD binding (Supplementary Tables 1–2). We further tested whether these antibodies could bind RBDs in VOCs (Fig. 5c and Supplementary Fig. 6). While two of the tested therapeutic antibodies are knocked out or exhibit reduced binding by mutations in Beta, six of seven are knocked out by Omicron and the other is substantially reduced in binding (23-fold). Importantly, the six ADI YYDRxG antibodies showed much less susceptibility to these variants with none being affected more than 3-fold by Beta and Delta (Fig. 5c and Supplementary Fig. 6) and five exhibiting between 3- and 16-fold reduction in RBD binding to Omicron BA.1 compared to ancestral wildtype SARS-CoV-2.

Our structural analysis of ADI-62113 interaction with the functionally conserved site of the RBD then provides the basis of broad neutralization of such YYDRxG antibodies (Fig. 1d, e). We next tested cross-neutralization of six ADI antibodies against SARS-CoV-2 VOCs and SARS-CoV. Using pseudotyped viruses, these YYDRxG antibodies were effective in neutralizing Alpha, Beta, Gamma, Delta VOCs, and two out of six neutralized Omicron BA.1 (ADI-62113 and ADI-62104) while others have weak to no neutralization (Fig. 5d and Supplementary Fig. 7). The reduction in maximal binding to Omicron RBD of ADI-63338, ADI-63164, ADI-57843, and ADI-62785 compared to ADI-62113 and ADI-62104 may explain their inability to neutralize this variant (Supplementary Fig. 6). Consistent with the sarbecovirus binding data, four of six cross-reactive ADI YYDRxG antibodies are able to also neutralize SARS-CoV with the most potent, ADI-62104, exhibiting an IC_50_ of 72 ng/ml. Combining with available cross-neutralization data for four other YYDRxG antibodies^11,16,36,47^, eight (36%) of these YYDRxG antibodies are able to cross-neutralize SARS-CoV. However, another 11 (50%) still need to be tested against SARS-CoV and may lead to an even higher frequency of cross-neutralization by these YYDRxG antibodies (Fig. 5a).

In addition, we analyzed antibodies from available neutralization data to see whether there was any preference in heavy chain or light chain variable gene usage paired with *IGHD3-22* that was correlated with broad and/or cross-neutralization. Several heavy and light chain V genes were more frequently used in neutralizing antibodies with fewer heavy chain genes represented than light chain genes. The combinations of heavy and light variable genes in neutralizing antibodies appear to be largely distinct from those in non-neutralizing antibodies (Fig. 5e). *IGHV3-30, IGHV3-33*, and *IGKV3-20* were found to encode the heavy and light chain variable domains in both neutralizing and non-neutralizing antibodies. Some limited preferences are seen for IGHV4-39 with IGKV1-17 and IGHV3-13 with IGLV2-14 or IGLV3-21 in neutralizing antibodies. However, the small number of bnAbs that have been experimentally verified to date (*n* = 22 neutralizing vs *n* = 6 non-neutralizing antibodies) preclude statistically meaningful analysis. Additional study of more YYDRxG antibodies will likely reveal whether heavy and light chain genes and CDR H3 length impact neutralization potency. Overall, our data suggest YYDRxG antibodies are broadly neutralizing against SARS-CoV-2 VOCs, and some against the Omicron variant. Furthermore, eight of 11 tested antibodies exhibit cross-neutralization activity to their sarbecoviruses (Fig. 5a right and Supplementary Table 1). Hence, the YYDRxG pattern encoded by *IGHD3-22* may represent a convergent solution by the human humoral system to counteract SARS-CoV-2 variants including Omicron, as well as other sarbecoviruses, if high enough binding affinity is attained and meets quite a stringent threshold compared to other viruses.

### YYDRxG motif-containing antibodies are frequently present in COVID-19 patients, vaccinees, and infected-then-vaccinated patients

**Fig. 6:**
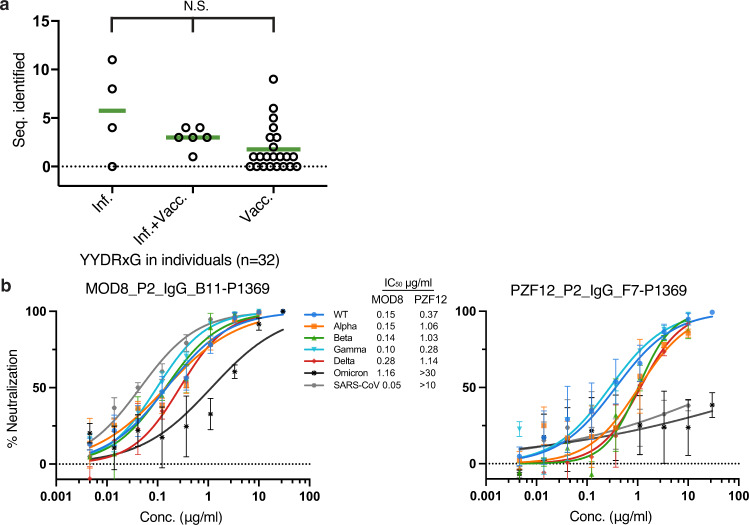
YYDRxG motif can potentially serve as a biomarker of broad neutralization against SARS-CoV-2 VOCs. **a** YYDRxG antibodies sequenced from COVID-19 and vaccinated individuals. The number of YYDRxG antibody sequences identified in each individual’s antibodies (Seq. identified) were split into three groups according to the medical history reported in their original publication^33–35,38,39,42^. All antibody sequences (*n* = 17,536) sequenced from COVID-19 patients without vaccination (*n* = 4), COVID-19 patients vaccinated post-recovery (*n* = 6), and vaccinees without COVID-19 history (*n* = 22) are included in this analysis. Non-parameter Kruskal-Wallis test was used to compare the number of YYDRxG antibodies isolated in each group. N.S., not significant (*P* > 0.05). Sequence counts for each individual are included in Supplementary Table 3. **b** Representative antibodies with only sequence information available show potent neutralization. MOD8_P2_IgG_B11-P1369 and PZF12_P2_IgG_F7-P1369, were selected from 72 sequences, which do not have any neutralization data available (Supplementary Table 2). The two antibodies were cloned, expressed, purified, and tested against SARS-CoV-2, VOCs and SARS-CoV in a pseudovirus neutralization assay. Error bars indicate standard deviation (SD) of at least two biological replicates. Neutralization potency, i.e., IC_50_s, are shown between the two panels.

Further analysis of 80 YYDRxG motif-containing antibodies sequenced from 32 donors^33–35,38,39^ suggests that this type of antibody can be frequently elicited in both infected and vaccinated individuals, as well as in COVID-19 patients who received mRNA vaccines post-recovery, although the abundance of such antibodies in serum is low (Fig. 6a and Supplementary Table 3). Antibodies such as C634, C652, C868, C996, C1243, C1332, C1381, C1463 have already showed potent neutralization to SARS-CoV-2 (Supplementary Table 1). Recently, C022 has been shown to neutralize all SARS-CoV-2 variants tested and bind the RBD in a similar manner^16^. Although many antibodies from these individuals have not been tested for neutralization against VOCs (Supplementary Tables 1–2), the strong correlation between the YYDRxG motif with experimentally characterized neutralizing antibodies suggest that these additional antibodies may broadly neutralize SARS-CoV-2 variants and potentially other sarbecoviruses (Fig. 5a and Supplementary Table 1). Indeed, we show here that two representative antibodies selected from the above cohort with only sequence information available, i.e., MOD8_P2_IgG_B11-P1369 and PZF12_P2_IgG_F7-P1369, exhibit broad neutralization against SARS-CoV-2 ancestral wildtype (WT), all VOCs, i.e., Alpha, Beta, Gamma, Delta, and Omicron, and SARS-CoV, although PZF12_P2_IgG_F7-P1369 has much lower potency against Omicron and SARS-CoV (Fig. 6b). Collectively, these data suggest YYDRxG antibodies present in these donors are contributing to broad protection against SARS-CoV-2 variants. Detecting the abundance of YYDRxG and homologous sequences in serum can potentially serve as a biomarker of neutralization breadth against SARS-CoV-2 variants and SARS-related viruses.

## Discussion

Overall, we report here on structural and functional characterization of a cross-neutralizing antibody, ADI-62113, to SARS-CoV-2 VOCs, including Omicron, and other sarbecoviruses. Despite differences in heavy chain and light chain variable germline gene usage, the binding mode of ADI-62113 to the SARS-CoV-2 RBD was similar to COVA1-16^11^. Based on this similarity, we determined that this particular binding mode appears to be determined primarily by a YYDRxG hexapeptide in CDR H3 that is encoded by *IGHD3-22*. The YYDRxG hexapeptide allows for extensive interaction with a highly conserved site on the RBD that was first identified as the epitope for antibody CR3022^28^. Furthermore, the YYDRxG region of the CDR H3 that is most intimately involved in RBD binding is stabilized by a β-bulge that defines the conformation of YYDRxG and adjacent residues that makes it a desirable feature for RBD recognition (Fig. 2 and Supplementary Fig. 2a). Using a YYDRxG pattern search, we were able to identify and verify many antibodies that potently neutralize SARS-CoV-2, VOCs, and SARS-CoV. We also note here that a portion of this motif, a YYD sequence, has been observed in bnAbs that target the HIV V2-apex^48,49^.

While RBS-targeting antibodies are highly potent for neutralizing specific SARS-CoV-2 viruses, most lack breadth against emerging variants and other sarbecoviruses^1,3–5,50–53^. However, broad and cross-neutralizing antibodies, albeit much rarer, have been identified against the highly conserved CR3022 site^11,28^. Key residues in this functionally conserved epitope site are involved in inter-protomer interactions between RBDs in the spike trimer and interaction between RBD and central helix and heptad repeat 1 in S2 subunit of the spike as we analyzed in our previous studies^11^. Mutation of these residues likely interrupt spike functionality, which may be the reason why mutations in this conserved site have rarely been so far in SARS-CoV-2 variants^14^. Several key epitope residues, such as F377, K378, and S383, are critical in RBD stability as revealed by previous deep mutational scanning data^54^. Hence, antibodies targeting this site are often broadly reactive to SARS-CoV-2 variants as well as to other sarbecoviruses. Nevertheless, mutations of apparently non-essential functional residues, e.g., S371L, S371F, and R408S, have been identified in Omicron BA.1 and BA.2. Several bnAbs, e.g., ADI-62113 (this study), 10–40^27^, and S2X259^13^, that have been structurally characterized to target this site effectively neutralize SARS-CoV-2 VOCs including Omicron BA.1. Even although their epitopes do not overlap with the receptor binding site, they can sterically compete with binding of human ACE2 receptor, which may in part explain their relatively high potency as non-RBS antibodies in neutralizing SARS-CoV-2 and VOCs.

High throughput methodologies especially single cell technologies and deep sequencing have advanced rapid antibody isolation to SARS-CoV-2^35,43,55–58^. Recently, repertoire sequencing has provided massive databases of antibody sequences^59,60^. However, experimental characterization such as cloning, expression, kinetics measurement, epitope binning, and structure characterization are more time-consuming and sometimes resource limiting for studying the antibody response to SARS-CoV-2 infection and vaccination. From a search of public human antibody sequences, we were able to identify 100 anti-SARS-CoV-2 antibodies containing a conserved YYDRxG motif exclusively encoded by the *IGHD3-22* gene (Fig. 3c and Supplementary Tables 1–2). A study reporting antibody 10–40^27^ showed that antibodies with a “YYDRSGY” motif that bind the CR3022 site are a subset within the larger family of antibodies that we identified here (Supplementary Fig. 4a and Supplementary Tables 1–2), further validating our strategy of combining YYDRxG homology motifs and CDR H3 length restriction in identification of bnAbs targeting the CR3022 site. The strong preference for one of the three possible reading frames of *IGHD3-22*, a somatic hypermutation at a specific position in this region, local structural constraints, and the requirement for an extended length of CDR H3, make these antibodies less abundant compared to some other neutralizing antibodies targeting SARS-CoV-2 RBD. Notwithstanding, our findings here illustrate that antibodies with a YYDRxG pattern in their CDR H3 have the capability to target a highly conserved surface in sarbecovirus RBDs and can neutralize SARS-CoV-2 VOCs including Omicron, and SARS-CoV, indicating a common convergent solution available to the human humoral immune system to counteract sarbecoviruses.

Finally, antibodies containing a YYDRxG feature can be elicited by both natural infection and vaccination but are in low abundance in serum. The high correlation between the presence of a YYDRxG pattern and broad neutralization activity suggest such antibodies may contribute to broad protection against SARS-CoV-2 variants, as well as other SARS-related coronaviruses. Several YYDRxG antibodies have shown effective in vivo protection against SARS-related viruses in animal models such as CC25.53^19^, CC25.54^19^, and 10–40^27^. Given the continuous mutation of SARS-CoV-2 in the current pandemic, broadly neutralizing antibodies targeting conserved sites, such as these YYDRxG antibodies, are urgently needed, especially when most clinical antibodies under emergency use authorization are knocked out by Omicron variants^6–9,61^. Vaccines that focus on the *IGHD3-22* gene and elicit YYDRxG antibodies may have increased breadth and prevent or ameliorate infection by SARS-CoV-2 variants and other sarbecoviruses. Interrogation of these signature sequences in serum can also serve as biomarkers to evaluate vaccine breadth and guide rational design of next-generation vaccines.

## Methods

### Expression and purification of SARS-CoV-2 RBD for crystallization

The receptor-binding domain (RBD) (residues 333–529) of the SARS-CoV-2 spike (S) protein (GenBank: QHD43416.1), was cloned into a customized pFastBac vector^62^, and fused with an N-terminal gp67 signal peptide and C-terminal His_6_ tag^28^. Recombinant bacmids encoding SARS-CoV-2 RBD were generated using the Bac-to-Bac system (Thermo Fisher Scientific) followed by transfection into Sf9 cells using FuGENE HD (Promega) to produce baculoviruses for RBD expression. RBD protein was expressed in High Five cells (Thermo Fisher Scientific) with suspension culture shaking at 110 r.p.m. at 28 °C for 72 h after the baculovirus transduction at an MOI of 5 to 10. Supernatant containing RBD protein was then concentrated using a 10 kDa MW cutoff Centramate cassette (Pall Corporation) followed by affinity chromatography using Ni-NTA resin (QIAGEN) and size exclusion chromatography using a HiLoad Superdex 200 pg column (Cytiva). The purified protein sample was buffer exchanged into 20 mM Tris-HCl pH 7.4 and 150 mM NaCl and concentrated for binding analysis and crystallographic studies.

### Expression and purification of antibodies

ADI-62113 IgG was produced in *S. cerevisiae* cultures, as used in previous study^26^. Yeast cultures were incubated at 30 °C and 80% relative humidity, with shaking at 650 rpm. Following 6 days of incubation, yeast cultures were cleared by centrifugation, and resultant supernatant containing IgG was purified by protein A chromatography. Bound IgGs were eluted from protein A resin using 200 mM acetic acid with 50 mM NaCl (pH 3.5) diluted into 1/8 [v/v] 2 M HEPES (pH 8.0) and subsequently buffer-exchanged into PBS (pH 7.0). Additional YYDRxG motif-containing monoclonal antibodies and antibodies under emergency use authorization were produced with the same method for characterization by sarbecovirus RBD yeast surface display, bio-layer interferometry, and neutralization assays. ADI-62113 Fab fragments for structural studies were generated by papain digestion for 2 hours at 30 °C, and the reaction was terminated by addition of iodoacetamide. Fab fragments were purified over Protein A resin to remove cleaved Fc fragments and undigested IgG. The flowthrough was then purified over CaptureSelect™ IgG-CH1 affinity resin (ThermoFisher Scientific), and bound Fab fragments eluted with 200 mM acetic acid with 50 mM NaCl (pH 3.5) diluted into 1/8 [v/v] 2 M HEPES (pH 8.0) before buffer exchanging into PBS (pH 7.0). Expression plasmids encoding the heavy (HC) and light chains (LC) of MOD8_P2_IgG_B11-P1369 and PZF12_P2_IgG_F7-P1369 were transiently co-transfected into ExpiCHO cells at a ratio of 2:1 (HC:LC) using ExpiFectamine™ CHO Reagent (Thermo Fisher Scientific) according to the manufacturer’s instructions. The supernatant was collected at 10 days post-transfection. The IgG antibodies and Fabs were purified with a CaptureSelect™ CH1-XL Matrix column (Thermo Fisher Scientific) for affinity purification and a HiLoad Superdex 200 pg column (Cytiva) for size exclusion chromatography. The purified Fab protein samples were buffer exchanged into 20 mM Tris-HCl pH 7.4 and 150 mM NaCl and concentrated for crystallographic studies and the IgG proteins used for binding and neutralization assays.

### YYDRxG pattern search

Over 205,000 antibody sequences including heavy and light chains were retrieved from GenBank using Biopython program^63^, supplemented with sequences reported in previous publications^33–35,38,39,42^, and then subjected to repertoire analysis using PyIR program^64^ implemented with IgBLAST^65^. CDR H3 amino-acid sequences from the compiled dataset were subjected to computational pattern search. Key residues in ADI-62113 and COVA1-16 interacting with SARS-CoV-2 RBD were analyzed using their crystal structures with SARS-CoV-2 RBD. YYDRxG and homologous sequences were used for the computational search. A length restriction with five or more amino acids prior and seven or more amino acids post YYDRxG key residues according to the structure analysis of ADI-62113 and COVA1-16 in complex with RBD was included in the search, which automatically yield a length restriction of >18 aa in CDR H3 that likely positions the YYDRxG motif to reach the RBD surface. This constraint was thus defined along with the YYDRxG pattern to search for matches in compiled CDR H3 datasets. Genbank accession identifiers of the resultant YYDRxG hits were used for retrieving the full sequence record from Genbank. The compiled data were analyzed manually with sequence check and literature curation. Sequences of the antibodies against SARS-CoV-2 were included in further analysis.

### Sequence analysis and surface conservation

Sequences of sarbecoviruses were retrieved from GenBank using Biopython program, aligned using MUSCLE program^66^ built in European Bioinformatics Institute (EBI) web services^67^ and scored for surface conservation in ConSurf server^68,69^. The conserved surface of SARS-CoV-2 RBD was visualized using the PyMOL program (Schrödinger, LLC). Alignment of the 169 sarbecovirus RBD sequences used for conservation analysis are listed in Supplementary Data 1. For visualization purpose, representative sarbecovirus RBD sequences from three clades were selected to compare with the conserved ADI-62113 epitope residues. BM48-31, YP_003858584; Rf1-2004, ABD75323; pCoV_GX-P5L, QIA48632; pCoV_GX-P2V, QIQ54048; RaTG13, QHR63300; SARS-CoV-2, YP_009724390; A021, QWQ56573; Rs4081, ATO98120; GD-pangolin, QLR06866; LYRa11, AHX37558; Civet010, AAU04649; CS24, ABF68959; HKU3, AAY88866; SARS-CoV, YP_009825051; Frankfurt 1, AAP33697; Rs4231, ATO98157; WIV1, AGZ48828; SHC014, QJE50589. Antibody sequences were also aligned using the same method and visualized using ESPript 3.0 server^70^.

### Crystallization and X-ray structure determination

The ADI-62113 Fab was mixed with SARS-CoV-2 RBD in an equimolar ratio and incubated overnight at 4 °C. 384 conditions of the JCSG Core Suite (Qiagen) were used for setting-up trays for screening at a concentration of 11.9 mg/ml on our robotic CrystalMation system (Rigaku) at Scripps Research. Crystallization trials were set-up by the vapor diffusion method in sitting drops containing 0.1 μl of protein complex and 0.1 μl of reservoir solution. Crystals appeared on day 2, were harvested on day 14, and flash cooled and stored in liquid nitrogen until data collection. Diffraction data were collected at cryogenic temperature (100 K) at the Stanford Synchrotron Radiation Lightsource (SSRL) on Scripps/Stanford beamline 12-1 with a beam wavelength of 0.97946 Å, and processed with HKL2000^71^. Diffraction data were collected from crystals grown in a drop containing 0.08 M sodium acetate pH 4.6, 0.16 M ammonium sulfate, 20% (w/v) polyethylene glycol 4000, and 20% (v/v) glycerol. The X-ray structure was solved by molecular replacement (MR) using PHASER^72^ with MR models for the RBD and Fab from PDB 7JMW^11^. Iterative model building and refinement were carried out in COOT^73^ and PHENIX^74^, respectively. Epitope and paratope residues, as well as their interactions, were identified with the PISA program^29^ using calculated buried surface area (BSA > 0 Å^2^) as the criterion.

### Binding to sarbecovirus RBDs via yeast surface display

To assess binding breadth, IgGs and recombinant human Fc-conjugated hACE2 (Sino Biological, 10108-H02H) were tested against the panel of 17 sarbecovirus RBDs expressed by yeast display as previously used^17^. EBY100 yeast were transformed with a plasmid encoding the yeast mating protein Aga2p linked to sarbecovirus RBD on the C terminus. To induce RBD expression, 0.5 OD_600_/ml of yeast were transferred to SGCAA media and cultured at 20 °C for 16–20 h with 180 rpm shaking. Next, IgGs and hACE2 were titrated via 3-fold serial dilutions from 100 nM to 0.5 pM. RBD-expressing cells were aliquoted into 96-well plates and incubated with 100 µl of 100 nM IgG for 30 min on ice. Next, cells were washed twice with PBSF (1× PBS, 0.1% BSA) before secondary detection with 1:100 dilutions of APC-conjugated mouse anti-hemagglutinin tag (HA).11 antibody (BioLegend, 901524), PE-conjugated goat anti-human IgG polyclonal antibodies (Southern Biotech, 2040-09), and propidium iodide (Invitrogen, P1304MP) for 20 min on ice. Cells were washed twice with PBSF before analyzing via flow cytometry on a BD FACS Canto II (BD Biosciences). Binding curves were fitted with four-parameter non-linear regression analysis to calculate the apparent equilibrium binding constant (*K*_D_^App^) in GraphPad Prism 9. Points exhibiting hook effects at higher concentrations were excluded from analysis.

### Binding to SARS-CoV-2 variant RBDs using Enzyme-linked immunosorbent assays (ELISA)

SARS-CoV-2 WT (Acro Biosystems, Cat #SPD-C52H3), Beta (Acro Biosystems, Cat #SPD-C52Hp), Delta (Acro Biosystems, Cat #SPD-C52Hh), and Omicron RBD (residues 319-537) (Acro Biosystems, Cat#SPD-C522e) of the SARS-CoV-2 spike protein were used for the binding assay. 96-well half-area clear-bottom plates (Costar) were coated with 25 μl per well of SARS-CoV-2 variant RBD proteins diluted to 5 μg/ml in PBS and incubated overnight at 4 °C. Wells were washed 3 times with PBS + 0.05% Tween-20 and then blocked with 3% BSA-PBS for 1 h at 37 °C. After removal of blocking buffer, serial dilutions of ADI YYDRxG or commercial antibodies dissolved in PBS + 1% BSA, 0.05% Tween-20 were added and incubated for 1 hour at 37 °C. Plates were then washed three times with PBS + 0.05% Tween-20 and then horseradish peroxidase (HRP)-conjugated anti-human IgG (Jackson ImmunoResearch, Cat#109-036-088) detection antibody was added at 1:5000 dilution in PBS + 1% BSA, 0.05% Tween-20 and incubated for 30 minutes at 37 °C. After washing three times with PBS + 0.05% Tween-20, 25 μl/well of 1-Step Ultra TMB-ELISA Substrate Solution (Thermo Fisher Scientific, Cat#34029) was added to detect binding followed by addition of an equal volume of stop reagent (2 M sulfuric acid). Absorbance was measured at 450 nm using a Spectramax microplate Reader (Molecular Devices).

### Pseudovirus production and neutralization assays

Plasmids encoding SARS-CoV, SARS-CoV-2, or other variants of the spike protein, with the ER retrieval signal removed were co-transfected with MLV-gag/pol and MLV-CMV-Luciferase plasmids into HEK293T cells to generate pseudoviruses. Lipofectamine 2000 (Thermo Fisher Scientific, 11668019) was used according to the manufacturer’s instructions. The cell culture supernatants containing S-pseudotyped MLV virions were collected at 48 h post transfection, filtered through a 0.22 μm membrane and stored at −80 °C until use. Lentivirus transduced Hela cells expressing hACE2 (GenBank: BAB40370.1) were enriched by fluorescence-activated cell sorting (FACS) using biotinylated SARS-CoV-2 RBD conjugated with streptavidin-Alexa Fluor 647 (Thermo, S32357). Stable cell lines with consistent and high hACE2 expression levels were established as HeLa-hACE2 and used in the pseudovirus neutralization assay. Monoclonal antibody IgGs were serially diluted with DMEM medium supplemented with 10% heat-inactivated FBS, 1% Q-max, and 1% P/S. 25 μl of 3-fold serial dilutions were incubated with 25 μl of pseudotyped viruses at 37 °C for 1 h in 96-well half-well plate (Corning, 3688). Right before the end of the incubation, HeLa-hACE2 cells were suspended with culture medium at a concentration of 2 × 10^5^/ml. The DEAE-dextran (Sigma, 93556-1 G) was added to the cell solutions at 20 μg/ml for enhanced infectivity. A total of 50 μl of the cell solution was distributed into each well. The supernatant was removed 48 h post incubation at 37 °C, and the neutralization efficiency was calculated by measuring the luciferase levels in the HeLa-hACE2 cells. Cells were washed and lysed in luciferase lysis buffer (25 mM Gly-Gly pH 7.8, 15 mM MgSO_4_, 4 mM EGTA, 1% v/v Triton X-100). After addition of Bright-Glo (Promega, PR-E2620) according to the manufacturer’s instruction, luminescence signal was measured in duplicate. At least two biological replicates were performed for neutralization assays with SARS-CoV-2, SARS-CoV-2 variants of concern, and SARS-CoV. The IgG half-maximal inhibitory concentration (IC_50_) values were calculated using four-parameter logistic regression (Hill equation) in GraphPad Prism 9. Data are shown as mean ± SD.

### Statistics and reproducibility

The pseudovirus neutralization data from at least two biological repeats were analyzed in Prism 9 software (GraphPad) using a four-parameter logistic regression model. Data are shown as mean ± SD. Data from individual subjects were analyzed with non-parametric Kruskal–Wallis test using Prism 9 software. *P* > 0.05 was considered as not significant.

### Reporting summary

Further information on research design is available in the Nature Research Reporting Summary linked to this article.

## Supplementary information

Supplementary Information

Description of Additional Supplementary Files

Supplementary Data 1

Supplementary Data 2

Reporting Summary

## Data Availability

The authors declare that the main data supporting the findings of this study are available within the article and its Supplementary Information. X-ray coordinates and structure factors are deposited in the RCSB Protein Data Bank under PDB ID: 7T7B for ADI-62113 in complex with SARS-CoV-2 RBD. Additional data that contributed to this study are present in the Supplementary Information and source data for sarbecovirus RBD sequence alignment, graphs and charts can be found in Supplementary Data 1–2 associated with this article. Correspondence and requests for materials and all other data should be addressed to Ian A. Wilson. ADI antibodies are available from Adimab (L.M.W.) with a completed Materials Transfer Agreement.

## Code availability

This study does not generate any custom code or mathematical algorithm that is deemed central to the conclusions.
