# Supplementary material for: Human antibodies to SARS-CoV-2 with a recurring YYDRxG motif retain binding and neutralization to variants of concern including Omicron
Source: Commun Biol. 2022 Jul 29;5:766. doi: 10.1038/s42003-022-03700-6 (PMC9336126; doi:10.1038/s42003-022-03700-6)
Supplement: Supplementary file 1 — Supplementary Information [file 42003_2022_3700_MOESM1_ESM.pdf]

Supplementary Information for

Human antibodies to SARS-CoV-2 with a recurring YYDRxG motif retain binding and neutralization to variants of concern including Omicron

Hejun Liu[1,¶], Chengzi I. Kaku[2,¶], Ge Song[3,¶], Meng Yuan[1], Raiees Andrabi[3], Dennis R. Burton[3,4], Laura M. Walker[2,5,*], Ian A. Wilson[1,6,*]

[1] Department of Integrative Structural and Computational Biology, The Scripps Research Institute, La Jolla, CA 92037, USA

[2] Adimab, LLC, Lebanon, NH 03766, USA.

[3] Department of Immunology and Microbiology, The Scripps Research Institute, La Jolla, CA 92037, USA

[4] Ragon Institute of MGH, MIT and Harvard, Cambridge, MA 02139, USA

[5] Adagio Therapeutics, Inc., Waltham, MA 02451, USA

[6] The Skaggs Institute for Chemical Biology, The Scripps Research Institute, La Jolla, CA, 92037, USA

¶ These authors contributed equally

* These authors jointly supervised this work

Correspondence: wilson@scripps.edu (I.A.W.), laura.walker@adimab.com (L.M.W.)

This PDF file includes:

- **Supplementary Figures 1-7**
- **Supplementary Tables 1-3**

Other Supplementary Information for this manuscript includes the following:

- **Supplementary Data 1-2**

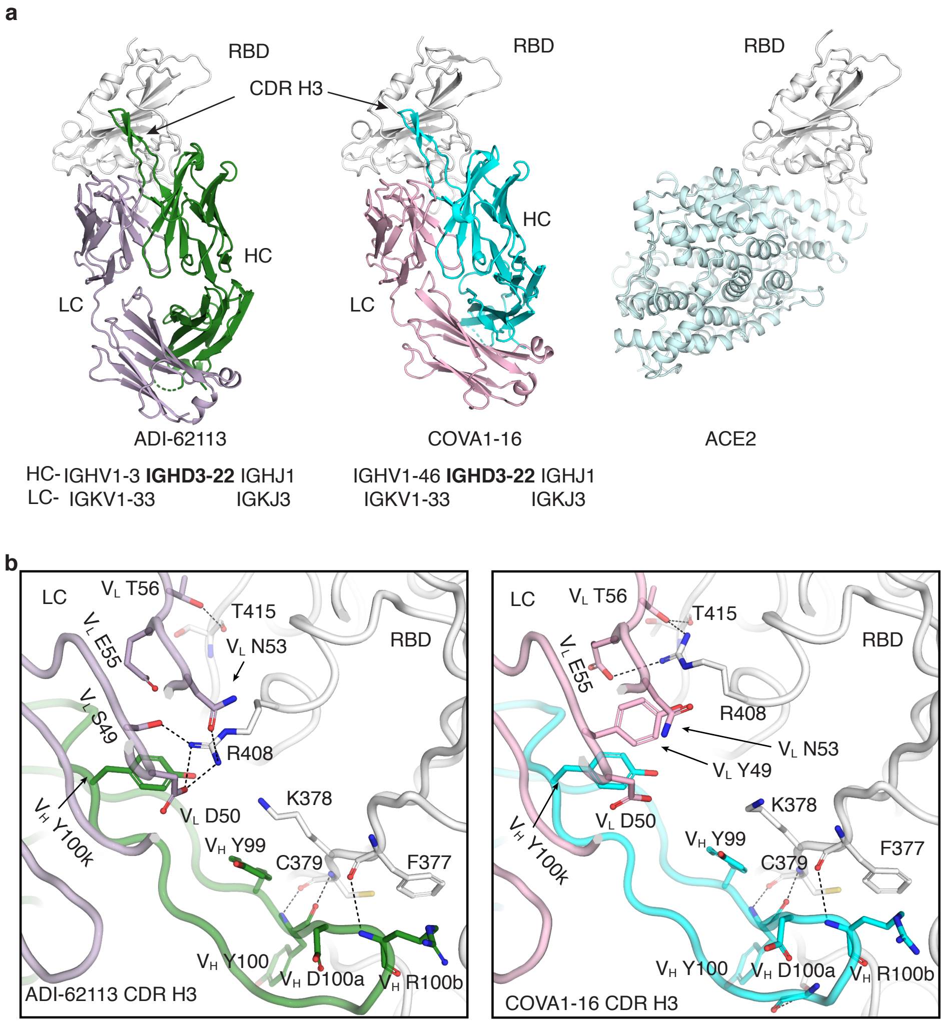

Supplementary Figure 1. Structural comparison of ADI-62113 and COVA1-16. a, A similar binding mode to SARS-CoV-2 RBD was found for ADI-62113 and COVA1-16. Structures are shown in ribbon representation. Both antibodies compete with ACE2 binding as shown in the far right even although their epitopes do not directly overlap with ACE2. SARS-CoV-2 RBD is shown in white. The same perspective view is used for all structures. The β-hairpin in CDR H3 is more twisted compared to the strands in the β-sheet in the core region of the Fab. Heavy chain (HC) and light chain (LC) immunoglobulin genes are shown under each antibody for comparison. Both antibodies use the *IGHD3-22* gene encoding the YYDRxG motif. **b,** Comparison between ADI-62113 and COVA1-16 Fab binding to SARS-CoV-2 RBD. ADI-62113 adopts a similar binding

mode to COVA1-16. A long CDR H3 containing the YYDRxG motif dominates the binding to SARS-CoV-2. Some differences in detailed interactions are seen due to sequence variation between the different antibodies, e.g., interaction with R408 of the RBD. The structures are shown in backbone tube representation with the side chains of residues involved in antibody-antigen interaction in sticks.

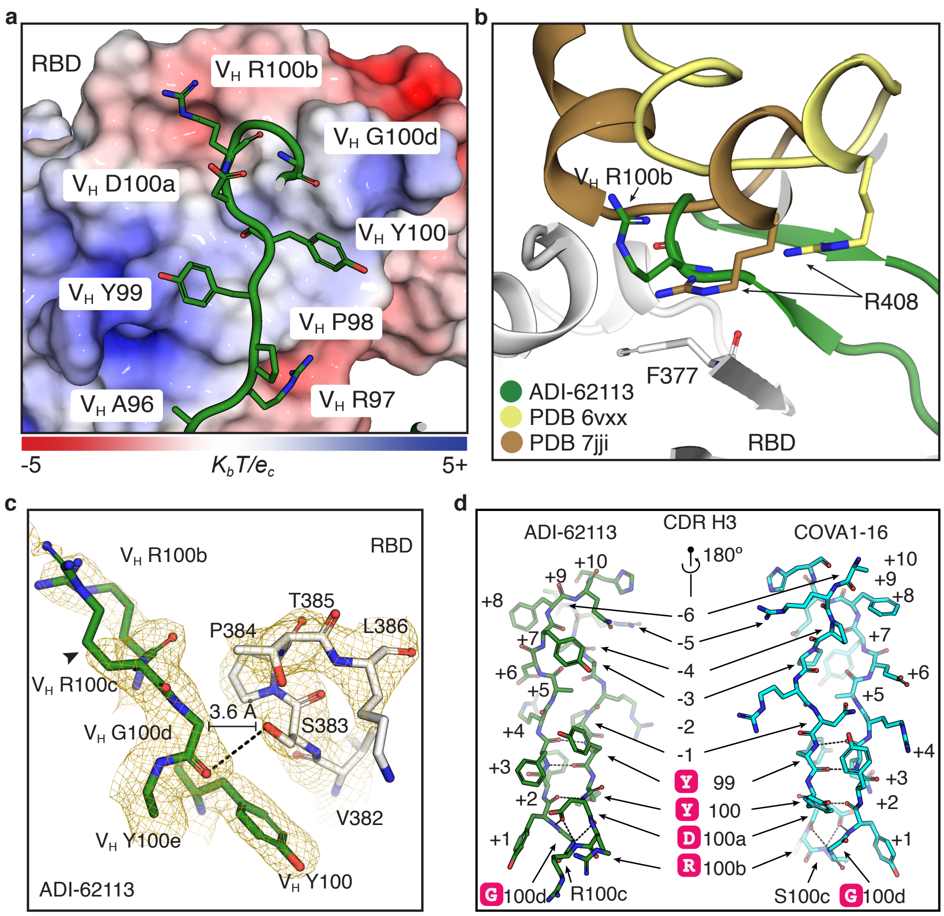

Supplementary Figure 2. YYDRxG motif analysis. a, CDR H3 of ADI-62113 bound to SARS-CoV-2 RBD surface. SARS-CoV-2 RBD is shown in an electrostatic surface representation. Red indicates a negatively charged surface and blue indicates a positively charged surface. The bar below the panel shows the electrostatic scale. CDR H3 is shown in a backbone tube representation with side chains in sticks. V_H R100b binds to a recessed surface on the RBD with an overall negative charge. **b,** The conserved surface where V_H R100b binds is also part of the interface between spike protomers when RBD is in the down conformation. Intra-protomer interaction among RBDs within a spike is variable due to slight differences among RBD conformations in different structures. Spikes with all RBD in “down” state are shown using two PDB IDs: 6VXX and 7JJI. R408 from the neighboring RBD can bind to the same site where V_H

R100b in ADI-62113 and COVA1-16 binds. Interaction between an arginine and F377 also seems to be common for both intra-protomer binding and antibody recognition at this site. **c.** Electron density around ADI-62113 V_H R100c. The electron density map is contoured at σ=1.0 using a 2mFo-DFc map calculated from the structure factors using PHENIX program[1]. The V_H R100c has poor to no side-chain density likely due to high mobility (indicated by an arrowhead). **d.** Comparison of the shared features of YYDRxG in ADI-62113 and COVA1-16. The hydrogen bonding observed with the YYDRxG hexapeptide is almost identical, indicating a conserved structure of the two CDR H3s. Sequences outside of YYDRxG are highly variable and two proline substitutions (at positions -3 and -4) have no impact on the YYDRxG conformation. The distance between YYDRxG to the first and last residue of CDR H3 seems important to position the hexapeptide at an appropriate distance at the tip of CDR H3 for RBD binding. A minimum of 5+ aa prior and 7+aa post YYDRxG hexapeptide should allow formation of a similar CDR H3 structure.

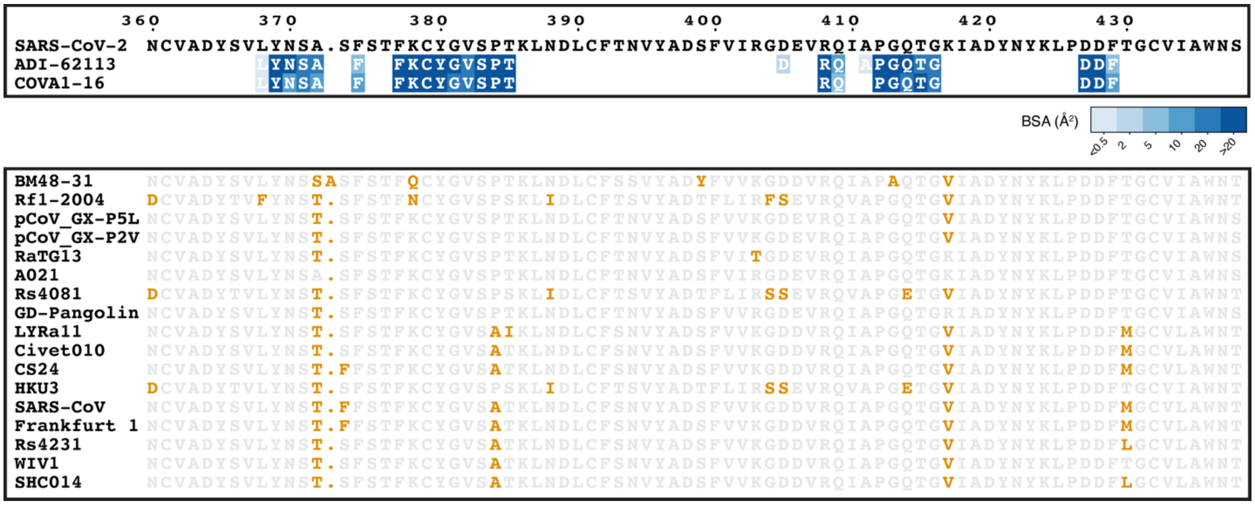

Supplementary Figure 3. Conserved epitope residues of YYDRxG antibodies. Epitope residues of ADI-62113 and COVA1-16, two YYDRxG antibodies, are highly conserved across sarbecoviruses. Epitope residues are highlighted on the sequence of SARS-CoV-2 RBD in the upper panel with blue tint indicating buried surface area (BSA). Epitope residues are defined by having the BSA by the indicated antibody larger than 0 Å^2. Other representative sarbecovirus sequences are aligned in the lower panel. Conserved residues to SARS-CoV-2 are shown in light grey and variable in orange. RBD amino-acid sequences were retrieved from GenBank database with the following IDs: BM48-31, YP_003858584; Rf1-2004, ABD75323; pCoV_GX-P5L, QIA48632; pCoV_GX-P2V, QIQ54048; RaTG13, QHR63300; SARS-CoV-2, YP_009724390; A021, QWQ56573; Rs4081, ATO98120; GD-Pangolin, QLR06866; LYRa11, AHX37558; Civet010, AAU04649; CS24, ABF68959; HKU3, AAY88866; SARS-CoV, YP_009825051; Frankfurt 1, AAP33697; Rs4231, ATO98157; WIV1, AGZ48828; SHC014, QJE50589. Alignment of all sarbecovirus RBDs are included in Supplementary Data 1.

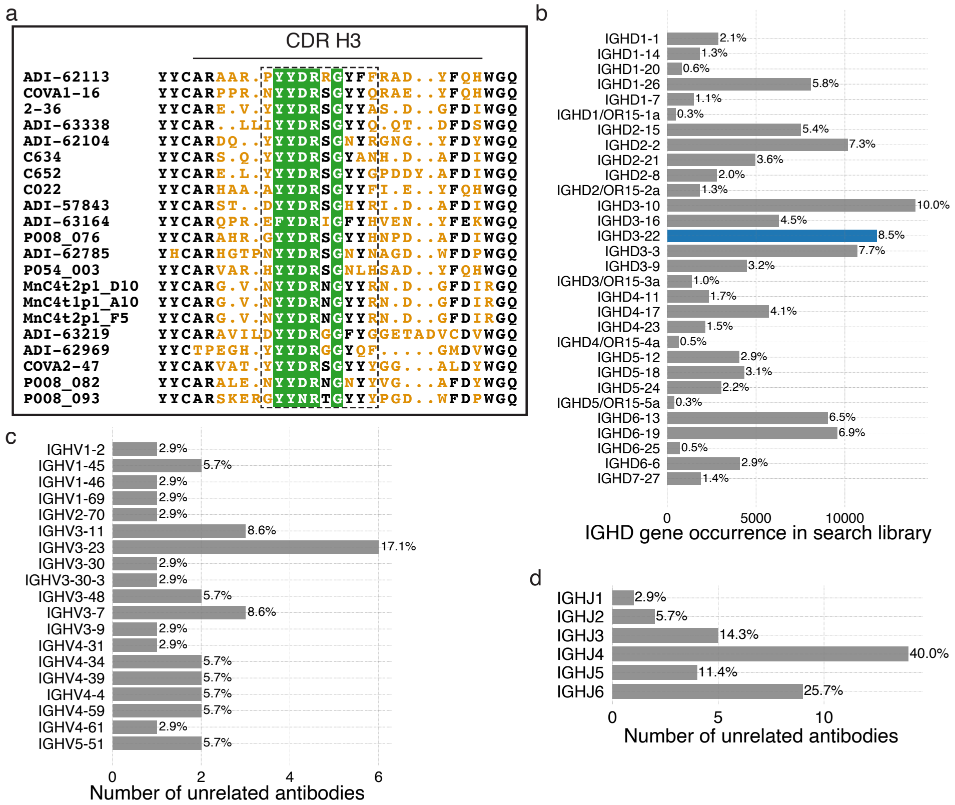

Supplementary Figure 4. YYDRxG antibodies identified from publicly available sequences. **a,** CDR H3 sequence alignment of representative YYDRxG antibodies against SARS-CoV-2. Conserved residues are shown in black and white, and variable residues in orange. Green background highlights key residues in the YYDRxG motif. CDR H3 sequences for all antibodies are included in Supplementary Table 2 (tested against SARS-CoV-2) and Supplementary Table 3 (sequence only). CDR H3 regions (IMGT definition) are indicated by the solid line above the sequences. Sequences encoded by *IGHD3-22* are boxed with a dashed line. **b,** Immunoglobulin diversity (IGHD) genes in the computational search library compiled with publicly available antibody sequences are described in Methods section. IGHD genes are diversely distributed in the overall library. No single IGHD gene is dominant in the antibody sequences, although some have higher propensity than others. *IGHD3-22* gene used to encode YYDRxG antibodies is colored blue. **c-d,** Immunoglobulin variable (IGHV) and joining (IGHJ) genes in YYDRxG

antibodies isolated from non-COVID-19 cohorts. The diverse sets of IGHV and IGHJ genes are distinct from, but partially overlap with, those observed in anti-SARS-CoV-2 antibodies, as shown in Fig. 3d-e.

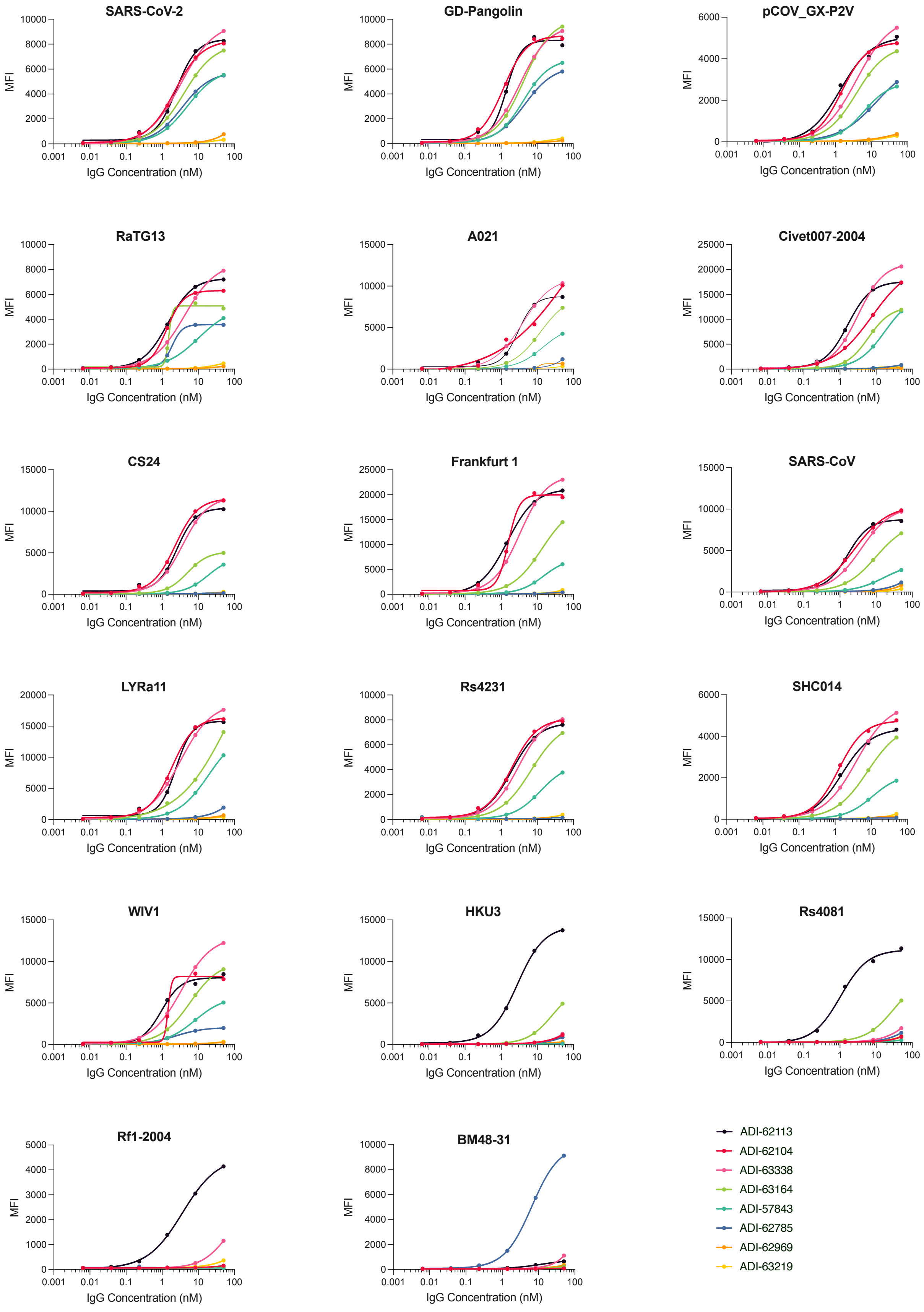
SARS-CoV-2
GD-Pangolin
pCOV_GX-P2V
RaTG13
A021
Civet007-2004
CS24
Frankfurt 1
SARS-CoV
LYRa11
Rs4231
SHC014
WIV1
HKU3
Rs4081
Rf1-2004
BM48-31
MFI
IgG Concentration (nM)
ADI-62113
ADI-62104
ADI-63338
ADI-63164
ADI-57843
ADI-62785
ADI-62969
ADI-63219

Supplementary Figure 5. Cross-reactivity of YYDRxG antibodies with sarbecoviruses. ADI antibodies were used for kinetic binding assay with sarbecovirus RBDs displayed on the yeast cell surface. Binding affinities are plotted as a heatmap in Fig. 5b. Most antibodies show potent binding to multiple sarbecoviruses, except for ADI-62969 and ADI-63219. The CDR H3 length of these two antibodies seems to be consistent with diminished binding to antigen.

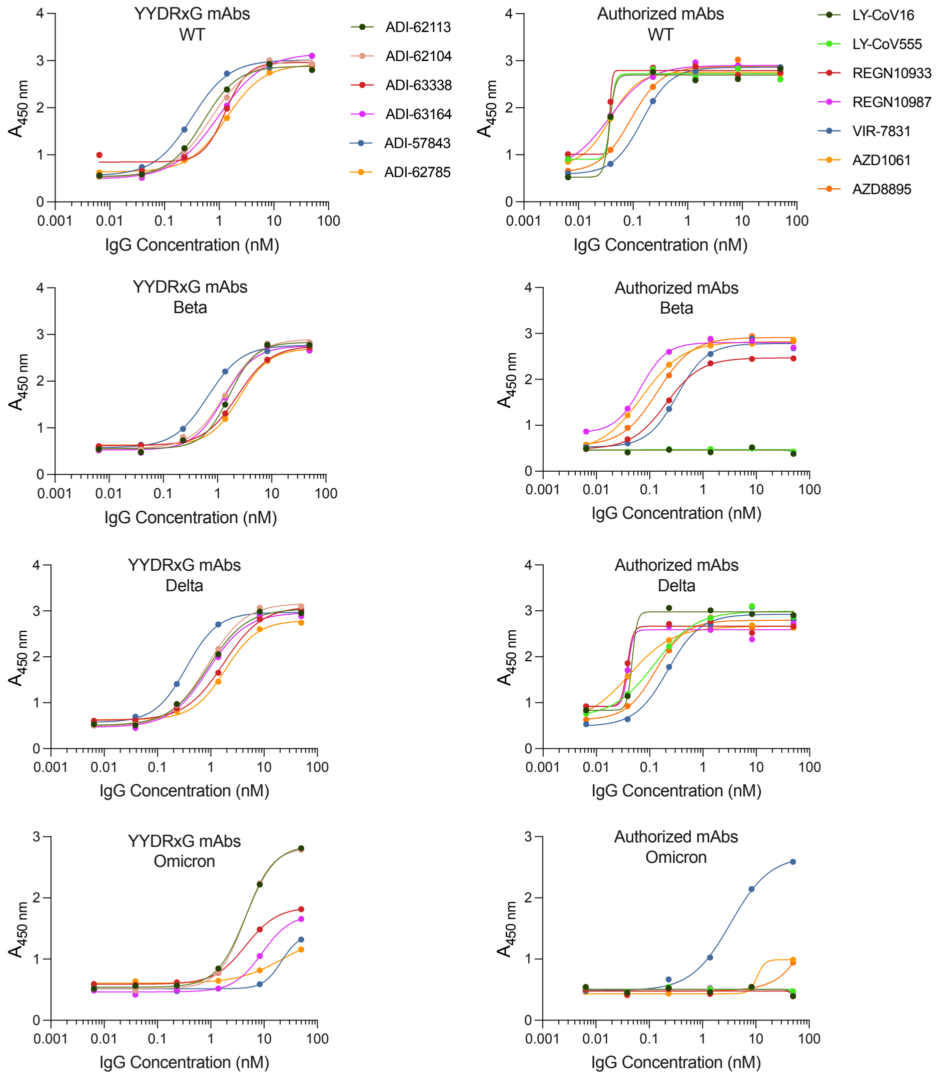

Supplementary Figure 6. Binding of YYDRxG antibodies to VOC RBDs. The Adimab YYDRxG antibodies generally retain their binding to more challenging variants, such as Omicron, Beta, and Delta, while almost all antibodies under emergency use authorization do not. The binding affinities, i.e., K_Ds, were determined in an enzyme-linked immunosorbent assay by titration of indicating antibodies. K_D changes compared to the ancestral strain (WT) are shown in Fig. 5c.

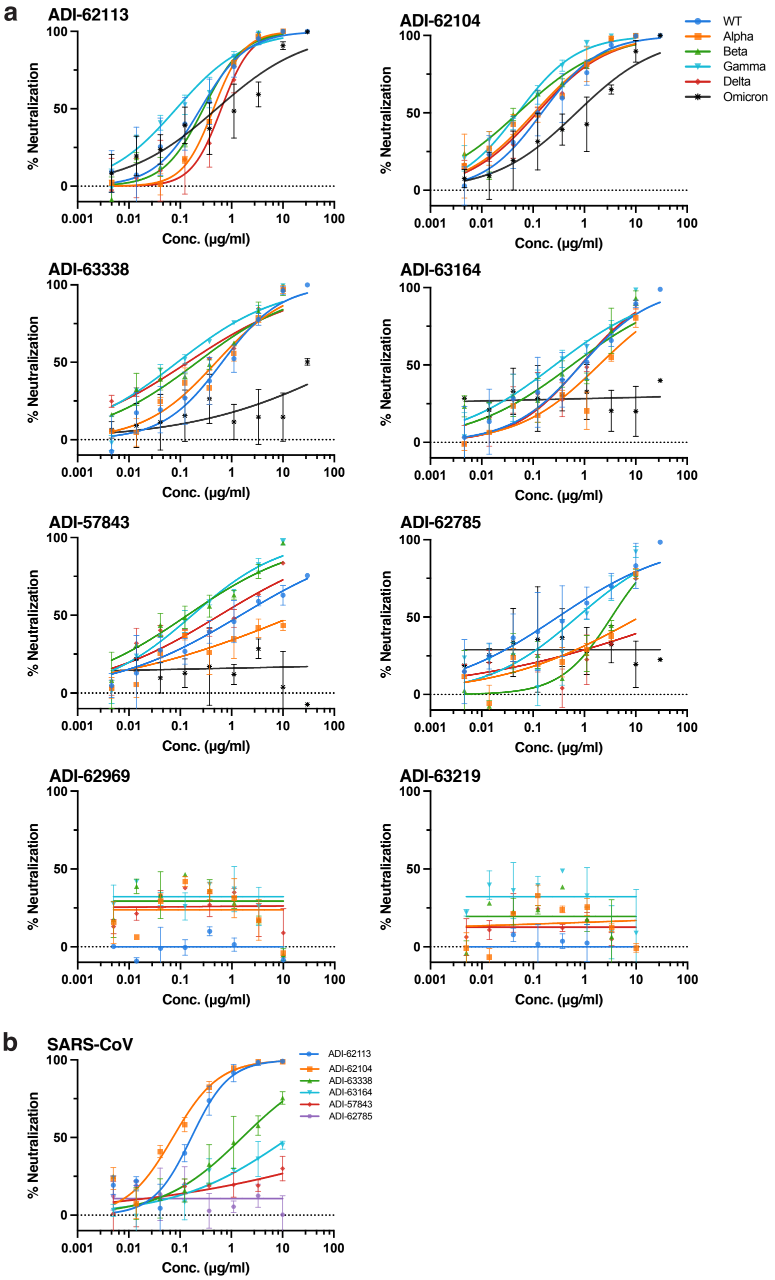

Supplementary Figure 7. YYDRxG antibodies neutralize SARS-CoV-2 VOCs and SARS-CoV. ADI antibodies were tested in a neutralization assay against SARS-CoV-2 wild type (WT), all the VOCs including Omicron, and SARS-CoV pseudoviruses. Neutralization potencies, IC_{50}s, are shown in Fig. 5d. Consistent with the binding kinetics assay, most antibodies broadly neutralize

SARS-CoV-2 variants (**a**) and SARS-CoV (**b**). Error bars indicate standard deviation (SD) of at least two biological replicates.

Supplementary Table 1. YYDRxG antibodies against SARS-CoV-2 with available neutralization data.

	Ab name	Neut.	Cr.	Epitope	CDR H3	Leng.	IGHV	IGHD	IGHJ	IGK/LV	PubMed ID
1	ADI-62113	Yes*	Yes*	RBD	ARAARPYYDRRGYFFRADYFQH	22	IGHV1-3	IGHD3-22	IGHJ1	IGKV1-33	33622975
2	COVA1-16	Yes	Yes	RBD	ARPPRNYYDRSGYYQRAEYFQH	22	IGHV1-46	IGHD3-22	IGHJ1	IGKV1-33	32540902
3	2-36	Yes	Yes	RBD	AREVYYYDRSGYYASDGFDI	20	IGHV4-61	IGHD3-22	IGHJ3	IGKV3-20	34836485
4	ADI-63338	Yes*	Yes*	RBD	ARLLIYYDRSGYYQQTDFDS	20	IGHV4-39	IGHD3-22	IGHJ4	IGLV6-57	33622975
5	ADI-62104	Yes*	Yes*	RBD	ARDQYYYDRSGNYRGNGYFDY	21	IGHV1-69	IGHD3-22	IGHJ4	IGKV1-33	33622975
6	C1463	Yes	N.D.	RBD	ARQLYYYDRSGFLHGDAFDI	20	IGHV3-30-3	IGHD3-22	IGHJ3	IGKV1-27	34619745
7	C996	Yes	N.D.	RBD	ARGQRGYYDRSGYYWGWRAFDI	22	IGHV3-13	IGHD3-22	IGHJ3	IGLV3-21	34126625
8	C634	Yes	N.D.	RBD	ARSQYYYDRSGYANHDAFDI	20	IGHV3-33	IGHD3-22	IGHJ3	IGKV1-5	33567448
9	C652	Yes	N.D.	RBD	ARELYYYDRSGYYGPDDYAFDI	22	IGHV3-33	IGHD3-22	IGHJ3	IGKV1-5	33567448
10	C868	Yes	N.D.	RBD	ARETFFYDRTGHYKSDGFDV	20	IGHV4-61	IGHD3-22	IGHJ3	IGKV1-5	34126625
11	C1381	Yes	N.D.	RBD	ARGPRGYYDRTGHFYRNWYFEL	22	IGHV3-13	IGHD3-22	IGHJ2	IGLV2-14	34619745
12	C022	Yes	Yes	RBD	ARHAAAYYDRSGYYFIEYFQH	21	IGHV4-39	IGHD3-22	IGHJ1	IGKV1-5	33948592
13	ADI-57843	Yes*	No*	RBD	ARSTDYYDRSGHYRIDAFDI	20	IGHV2-26	IGHD3-22	IGHJ3	IGKV3D-20	33622975
14	ADI-63164	Yes*	Yes*	RBD	ARQPREFYDRIGFYHVENYFEK	22	IGHV1-3	IGHD3-22	IGHJ4	IGKV3-20	33622975
15	P008_076	Yes	Yes	Spike	ARAHRGYYDRSGYYHNPDAFDI	22	IGHV3-13	IGHD3-22	IGHJ3	IGLV2-14	33836142
16	ADI-62785	Yes*	No*	RBD	ARHGTPNYYDRSGNYNAGDWFDP	23	IGHV4-39	IGHD3-22	IGHJ5	IGLV1-40	33622975
17	P054_003	Yes	No	RBD	ARVARHYYDRSGNLHSADYFQH	22	IGHV1-2	IGHD3-22	IGHJ1	IGKV1-33	33836142
18	MnC4t2p1_D10	Yes	N.D.	RBD	ARGVNYYDRNGYYRNDGFDI	20	IGHV4-39	IGHD3-22	IGHJ3	IGKV1-17	32673567
19	MnC4t1p1_A10	Yes	N.D.	RBD	ARGVNYYDRNGYYRNDGFDI	20	IGHV4-39	IGHD3-22	IGHJ3	IGKV1-17	32673567
20	MnC4t2p1_F5	Yes	N.D.	RBD	ARGVNYYDRNGYYRNDGFDI	20	IGHV4-39	IGHD3-22	IGHJ3	IGKV1-17	32673567
21	C1243	Yes	N.D.	RBD	AKDGGPYYYDRSGYARDDYYGMDV	24	IGHV3-30	IGHD3-22	IGHJ6	IGLV1-40	34619745
22	C1332	Yes	N.D.	RBD	AKAPRGYYDRSGYYRIQDNFDY	22	IGHV3-30	IGHD3-22	IGHJ4	IGKV1-13	34619745
23	ADI-63219	No*		spike	ARAVILDYYDRGGFYGGETADVCDV	25	IGHV1-18	IGHD3-22	IGHJ6	IGKV3-20	33622975
24	ADI-62969	No*		spike	TPEGHYYYDRGGYQFGMDV	19	IGHV3-30	IGHD3-22	IGHJ6	IGKV2-30	33622975
25	COVA2-47	No		Spike	AKVATYYYDRSGYYYGGALDY	21	IGHV3-9	IGHD3-22	IGHJ4	IGKV4-1	32540902
26	P008_082	No		RBD	ARALENYYDRNGNYYVGAFDY	21	IGHV1-18	IGHD3-22	IGHJ4	IGKV3-20	33836142
27	P008_093	No		RBD	ARSKERGYYNRTGYYYPGDWFDP	23	IGHV1-8	IGHD3-22	IGHJ5	IGKV3-11	33836142
28	C83A5	No		RBD	ARDGAYYDRSAYYYRLKFYFDF	22	IGHV3-33	IGHD3-22	IGHJ4	IGLV3-25	34332650

Neut., neutralization against SARS-CoV-2; Cr., cross neutralization against both SARS-CoV-2 and SARS-CoV. Leng., amino acid length of CDR H3 as defined by IMGT scheme. YYDRxG antibodies tested against SARS-CoV-2 or SARS-CoV in previous publications or in this study are included in this table. *neutralization data reported in this study. CDR H3 sequences, as well as heavy variable (IGHV), diversity (IGHD), joining (IGHJ) genes, and light chain variable genes (IGKV, IGLV) that encode these antibodies, are shown.

Supplementary Table 2. YYDRxG antibodies against SARS-CoV-2 with only sequences available.

	Ab name	CDR H3	Leng.	IGHV	IGHD	IHHJ	PubMed ID
1	COV021_P1_IgA_48-P1609	AKAALRGYYDRSGYYYENYNGMDV	24	IGHV3-30	IGHD3-22	IGHJ6	33288661
2	COVD21_P2_HC_H11-p1369	ARHAAAYYDRSGYYFIEYFQH	21	IGHV4-39	IGHD3-22	IGHJ1	33288661
3	COV021_P3_IgA_29-P1609	AKAALRGYYDRSGYYYENYNGMDV	24	IGHV3-30	IGHD3-22	IGHJ6	33288661
4	COVD21_P2_HC_H4-p1369	ARQWRGYYDRSGYYHFDAFDI	21	IGHV5-51	IGHD3-22	IGHJ3	33288661
5	COVD21_P3_HC_C11-p1369	ARVGYYYDRSGFPRTEDYFDY	21	IGHV1-69	IGHD3-22	IGHJ4	33288661
6	COV047_P5_IgG_15-P1369	ARYTYYYDRSGYYRPDYFDY	20	IGHV1-69	IGHD3-22	IGHJ4	33288661
7	COV047_P3_IgG_77-P1369	ARVPRGYYDRSGYYYLPHYLDY	22	IGHV1-18	IGHD3-22	IGHJ4	33288661
8	COV096_HC_72-p1369	ARRPRDYYDRSGYYYVPGYFDY	22	IGHV1-18	IGHD3-22	IGHJ4	33288661
9	COV107_Plate2_HC_47-P1369	TRGSRGYYDRSGYYTPLDPYYGMDV	25	IGHV3-21	IGHD3-22	IGHJ6	33288661
10	MOD3_P1_IgG_C3-P1369	ARDRGYFNRTGNYYVGRFDP	20	IGHV3-33	IGHD3-22	IGHJ5	33567448
11	Mod4_P3_IgG_H4-P1369	AKDWSAYYYDRSGYLRLDYYYGMDV	25	IGHV3-23	IGHD3-22	IGHJ6	33567448
12	Mod4_P3_IgG_H6-P1369	ARPELGYFDRSGYYREGEFFDY	22	IGHV3-33	IGHD3-22	IGHJ4	33567448
13	Mod4_P4_IgG_G8-P1369	ARTLYYYDRSGYGQSDDAFDI	21	IGHV3-74	IGHD3-22	IGHJ3	33567448
14	MOD8_P2_IgG_H4-P1369	ARQEHYYDRSGYPRSDYAFEI	21	IGHV4-39	IGHD3-22	IGHJ3	33567448
15	MOD8_P2_IgG_G11-P1369	ARSQNYYDRSGSLAEDAFDI	20	IGHV3-33	IGHD3-22	IGHJ3	33567448
16	MOD8_P2_IgG_B11-P1369	ARSQNYYDRSGYLAEDAFDI	20	IGHV3-33	IGHD3-22	IGHJ3	33567448
17	MOD8_P2_IgG_E11-P1369	ARHEYYYDRRGYPRLGWFDP	20	IGHV4-39	IGHD3-22	IGHJ5	33567448
18	PZF12_P2_IgG_F7-P1369	ARSPRGYYDRTGYYHLEYYFDY	22	IGHV3-30-3	IGHD3-22	IGHJ4	33567448
19	C001_P2_IgG_F6-P1369	ARDPFYYDRSGSPRGDAFDI	20	IGHV3-33	IGHD3-22	IGHJ3	33567448
20	C003_P1_IgG_C3-P1369	ARETWYYDRSGYPRSDEYFQY	21	IGHV1-69	IGHD3-22	IGHJ1	33567448
21	COV2-vax-3	ARVVPNYFDRSGYYPGAFDI	20	IGHV3-21	IGHD3-22	IGHJ3	34411541
22	COVD21_mo6_HC_P2D12-p1369	ARVPRDYYDRTGNHHVDEYFQH	22	IGHV3-74	IGHD3-22	IGHJ1	34126625
23	COVD21_mo6_HC_P1F9-p1369	ARESFYYDRSGYYGSDAFDI	20	IGHV4-61	IGHD3-22	IGHJ3	34126625
24	COV21_1y_P1_IgG_A10-P1369	ARETYYYDRSGYYSSDGFDY	20	IGHV4-61	IGHD3-22	IGHJ4	34126625
25	COV21_1y_P1_IgG_C1-P1369	ARETFYYDRTGYYSSDGFDF	20	IGHV4-61	IGHD3-22	IGHJ4	34126625
26	COVD47_mo6_P1_HC_B1-p1369	ATELYYYDRSGFQHWDGFAI	20	IGHV4-39	IGHD3-22	IGHJ3	34126625
27	COVD47_mo6_P1_HC_G7-p1369	ARDMEVDYYDRSGHYHVFHAFDI	23	IGHV3-30	IGHD3-22	IGHJ3	34126625
28	COV47_1y_P2_IgG_B9-P1369	ARRALGDYYDRTGYYHRLGFDS	22	IGHV5-51	IGHD3-22	IGHJ4	34126625
29	COV47_1y_P1_IgG_G5-P1369	ARRALGDYYDRHGHFHRLGFDY	22	IGHV5-51	IGHD3-22	IGHJ4	34126625
30	COV47_1y_P2_IgG_D12-P1369	ARHERSYYDRSGYYERRTLNDY	22	IGHV5-10-1	IGHD3-22	IGHJ4	34126625
31	COV47_1y_P2_IgG_B11-P1369	AKEPRGYYDRSGDYYILPPDF	21	IGHV3-23	IGHD3-22	IGHJ4	34126625
32	COV57_1y_P2_IgG_A10-P1369	ARGGRYYYDRSGYLKSDDVFDF	22	IGHV1-3	IGHD3-22	IGHJ3	34126625
33	COV57_1y_P2_IgG_E7-P1369	ARGQRAYYNRTGHYWGWRALDI	22	IGHV3-13	IGHD3-22	IGHJ3	34126625
34	COV107_6mo_P1_IGG_F9-P1369	ARPPRYYYDRSGYYVWEDYFDY	22	IGHV5-10-1	IGHD3-22	IGHJ4	34126625
35	COV107_6mo_P1_IGG_E11-P1369	ARVPRGYYDRSGHYHGQDYFDY	22	IGHV1-3	IGHD3-22	IGHJ4	34126625
36	COV107_6mo_P1_IGG_B9-P1369	ARLLHYYDRSGYSGATDDAFDI	22	IGHV5-51	IGHD3-22	IGHJ3	34126625
37	COV134_6mo_P1_IGG_B2-P1369	ARHSAHYYDRSGKFHWGYLHR	21	IGHV4-39	IGHD3-22	IGHJ1	34126625
38	COV134_6mo_P1_IGG_B6-P1369	ARTPDYYDRSGYLQTGSWFDP	21	IGHV1-18	IGHD3-22	IGHJ5	34126625
39	COV134_6mo_P1_IGG_H8-P1369	ARDLYYYDRSGNYNLEEYFQQ	21	IGHV1-69	IGHD3-22	IGHJ1	34126625
40	COV134_6mo_P1_IGG_D5-P1369	ARGSANYYDRHGHYYVASFLAN	22	IGHV1-69	IGHD3-22	IGHJ4	34126625
41	COV135_6m_P2_IgG_D9-P1369	ARDVYYYDRSGYLRFDFDN	19	IGHV4-39	IGHD3-22	IGHJ4	34126625
42	COV135_1y_P2_IgG_E9-P1369	ARPPRDYYERSGYYRFADYFQH	22	IGHV3-7	IGHD3-22	IGHJ1	34126625
43	COV135_1y_P2_IgG_F2-P1369	AKVSSPNYYDRSGKVRSEREDAFDI	25	IGHV3-23	IGHD3-22	IGHJ3	34126625
44	COV325_1m_P3_IgG_C3-P1369	TRDLRAYYDRSGYYVYGIGTDY	22	IGHV1-46	IGHD3-22	IGHJ4	34126625
45	COV325_6m_P2_IgG_H9-P1369	ARASGINYYDRSGNYNVDYYHYGMDV	26	IGHV3-33	IGHD3-22	IGHJ6	34126625
46	COV325_1y_P1_IgG_E7-P1369	AREVYYYDRRGYYKSEGFDH	20	IGHV4-61	IGHD3-22	IGHJ4	34126625
47	COV325_6m_P1_IgG_C11-P1369	ARDHIASYYDRSGHYHYYGMDV	22	IGHV1-69	IGHD3-22	IGHJ6	34126625
48	COV539_1m_P3_IgG_F12-P1369	ARRPRGYYDRSGYYILEDYFEY	22	IGHV3-33	IGHD3-22	IGHJ4	34126625
49	COV539_1m_P2_IgG_C7-P1369	ARHEYYYDRRGYSRLGWFDP	20	IGHV4-39	IGHD3-22	IGHJ5	34126625
50	COV539_6m_P2_IgG_C10-P1369	ARRPRGYYDRSGFYNVEDYFDY	22	IGHV3-33	IGHD3-22	IGHJ4	34126625
51	C006_mo6_HC_P4D5-1369	ARAGRDYYDRSGYHGHDAFDI	21	IGHV3-53	IGHD3-22	IGHJ3	34619745
52	C012_V1_P1_IgG_E1-P1369	ARSPRGYYDRSGYLNLLWYFDY	22	IGHV3-33	IGHD3-22	IGHJ4	34619745
53	C012_VAX2_HC_P1C11-1369	AREPRDYYDRSGYHRFLHYFEY	22	IGHV3-48	IGHD3-22	IGHJ4	34619745
54	C012_mo6_HC_P4A3-1369	ARRPRGYYDRSGYYHSIGYMDV	22	IGHV3-30	IGHD3-22	IGHJ6	34619745
55	C012_mo6_HC_P3H8-1369	ARRPRGYYDRSGYYHSIGYMDV	22	IGHV3-30	IGHD3-22	IGHJ6	34619745
56	C012_mo6_HC_P3E12-1369	ARRPRGYYDRSGYYHSIGYMDV	22	IGHV3-30	IGHD3-22	IGHJ6	34619745
57	C012_mo6_HC_P3G1-1369	ARRPRGYYDRSGYYHSIGYMDV	22	IGHV3-30	IGHD3-22	IGHJ6	34619745
58	C012_mo6_HC_P1E5-1369	ARRPRGYYDRSGYYHSIGYMDV	22	IGHV3-30	IGHD3-22	IGHJ6	34619745
59	C012_mo6_HC_P1E7-1369	ARVNYYYDRSGQPTLDDIFNI	21	IGHV1-69	IGHD3-22	IGHJ3	34619745
60	C012_mo6_HC_P2B2-1369	ARFGAYYDRTGYKKFEYFQH	20	IGHV5-51	IGHD3-22	IGHJ1	34619745
61	C016_mo6_HC_P2F10-1369	ARRPRGYYDRSGYYHSIGYMDV	22	IGHV3-30	IGHD3-22	IGHJ6	34619745
62	C016_mo6_HC_P1B9-1369	ARRPRGYYDRSGYYHSIGYMDV	22	IGHV3-30	IGHD3-22	IGHJ6	34619745
63	C016_mo6_HC_P1C2-1369	ARALCYYDRSGNYLTEDYFDH	21	IGHV1-3	IGHD3-22	IGHJ4	34619745
64	C017_Vax2_HC_P2H8-1369	ASCRYYYDRSGYAQPEDYFDY	21	IGHV1-69	IGHD3-22	IGHJ4	34619745
65	C003_6m_P1_IgG_H7-P1369	AKVGYYFDRNGSPRDENYYYYGMDV	25	IGHV3-30	IGHD3-22	IGHJ6	34619745
66	C003_6m_P3_IgG_H6-P1369	ARDVPLYYDRSGNYGLAEYFQY	22	IGHV1-18	IGHD3-22	IGHJ1	34619745
67	C003_6m_P3_IgG_C12-P1369	ARSPRDYYDRRGQIVVERWFDP	22	IGHV4-59	IGHD3-22	IGHJ5	34619745
68	C003_mo6_HC_P7A9-1369	ARRPRGYYDRSGYYHSIGYMDV	22	IGHV3-30	IGHD3-22	IGHJ6	34619745
69	C004_mo6_HC_P4E5-1369	ARAGRDYYDRSGYHGHDAFDI	21	IGHV3-53	IGHD3-22	IGHJ3	34619745
70	C004_mo6_HC_P4A3-1369	ARRPRGYYDRSGYYHSIGYMDV	22	IGHV3-30	IGHD3-22	IGHJ6	34619745
71	C004_mo6_HC_P3F4-1369	ARRPRGYYDRSGYYHSIGYMDV	22	IGHV3-30	IGHD3-22	IGHJ6	34619745
72	C004_mo6_HC_P4F5-1369	ARRPRGYYDRSGYYHSIGYMDV	22	IGHV3-30	IGHD3-22	IGHJ6	34619745

YYDRxG antibodies against SARS-CoV-2 with no available antigen binding or neutralization data are included in this table. CDR H3 sequences, as well as heavy variable (IGHV), diversity (IGHD), and joining (IGHJ) genes that encode these antibodies, are shown.

Supplementary Table 3. YYDRxG antibodies sequenced from COVID-19 patients and vaccinees.

	Patient ID	Sequence analyzed	YYDRxG found	Patient group
1	COV021	1689	11	Infection
2	COV047	1346	8	Infection
3	COV072	1031	0	Infection
4	COV107	1376	4	Infection
5	COV057	739	3	Infection+vaccination
6	COV096	1331	1	Infection+vaccination
7	COV134	515	4	Infection+vaccination
8	COV135	531	3	Infection+vaccination
9	COV325	708	4	Infection+vaccination
10	COV539	778	3	Infection+vaccination
11	C001	556	1	Vaccination
12	C003	536	6	Vaccination
13	C004	592	5	Vaccination
14	C005	215	1	Vaccination
15	C006	326	1	Vaccination
16	C011	526	0	Vaccination
17	C012	683	9	Vaccination
18	C016	742	3	Vaccination
19	C017	421	1	Vaccination
20	C030	224	1	Vaccination
21	C034	343	0	Vaccination
22	MOD1	136	0	Vaccination
23	MOD11	218	0	Vaccination
24	MOD2	89	1	Vaccination
25	MOD3	233	2	Vaccination
26	MOD4	245	3	Vaccination
27	MOD6	220	0	Vaccination
28	MOD7	218	0	Vaccination
29	MOD8	288	4	Vaccination
30	MOD9	203	0	Vaccination
31	PZF10	263	0	Vaccination
32	PZF12	180	1	Vaccination

A YYDRxG pattern search was used to identify antibody sequences in previously reported cohorts[2-7]. Number of all antibody sequences analyzed and YYDRxG antibodies identified in each patient were counted and used for analysis in Fig. 6a.

Supplementary References

1 Adams, P. D. *et al.* PHENIX: a comprehensive Python-based system for macromolecular structure solution. *Acta Crystallogr D* **66**, 213-221 (2010).

2 Cho, A. *et al.* Anti-SARS-CoV-2 receptor-binding domain antibody evolution after mRNA vaccination. *Nature* **600**, 517-522 (2021).

3 Wang, Z. *et al.* Enhanced SARS-CoV-2 neutralization by dimeric IgA. *Sci Transl Med* **13**, eabf1555 (2021).

4 Wang, Z. *et al.* mRNA vaccine-elicited antibodies to SARS-CoV-2 and circulating variants. *Nature* **592**, 616-622 (2021).

5 Wang, Z. *et al.* Naturally enhanced neutralizing breadth against SARS-CoV-2 one year after infection. *Nature* **595**, 426-431 (2021).

6 Gaebler, C. *et al.* Evolution of antibody immunity to SARS-CoV-2. *Nature* **591**, 639-644 (2021).

7 Robbiani, D. F. *et al.* Convergent antibody responses to SARS-CoV-2 in convalescent individuals. *Nature* **584**, 437-442 (2020).
