# Supplementary material for: Human antibodies to SARS-CoV-2 with a recurring YYDRxG motif retain binding and neutralization to variants of concern including Omicron
Source: Commun Biol. 2022 Jul 29;5:766. doi: 10.1038/s42003-022-03700-6 (PMC9336126; doi:10.1038/s42003-022-03700-6)
Supplement: Supplementary file 2 — Description of Additional Supplementary Files [file 42003_2022_3700_MOESM2_ESM.pdf]

Description of Additional Supplementary Files

File name: Supplementary Data 1
Description: Amino acid sequence alignment of sarbecovirus RBDs.

File name: Supplementary Data 2
Description: The source data behind the graphs in the paper.
