# Supplementary material for: Human antibodies to SARS-CoV-2 with a recurring YYDRxG motif retain binding and neutralization to variants of concern including Omicron
Source: Commun Biol. 2022 Jul 29;5:766. doi: 10.1038/s42003-022-03700-6 (PMC9336126; doi:10.1038/s42003-022-03700-6)
Supplement: Supplementary file 5 — Reporting Summary [file 42003_2022_3700_MOESM5_ESM.pdf]

nature portfolio

Corresponding author(s): Ian A. Wilson

Last updated by author(s): Feb 28, 2022

Reporting Summary

Nature Portfolio wishes to improve the reproducibility of the work that we publish. This form provides structure for consistency and transparency in reporting. For further information on Nature Portfolio policies, see our Editorial Policies and the Editorial Policy Checklist.

Statistics

For all statistical analyses, confirm that the following items are present in the figure legend, table legend, main text, or Methods section.

n/a	Confirmed	
☐	☒	The exact sample size (*n*) for each experimental group/condition, given as a discrete number and unit of measurement
☐	☒	A statement on whether measurements were taken from distinct samples or whether the same sample was measured repeatedly
☐	☒	The statistical test(s) used AND whether they are one- or two-sided<br>*Only common tests should be described solely by name; describe more complex techniques in the Methods section.*
☒	☐	A description of all covariates tested
☒	☐	A description of any assumptions or corrections, such as tests of normality and adjustment for multiple comparisons
☐	☒	A full description of the statistical parameters including central tendency (e.g. means) or other basic estimates (e.g. regression coefficient) AND variation (e.g. standard deviation) or associated estimates of uncertainty (e.g. confidence intervals)
☐	☒	For null hypothesis testing, the test statistic (e.g. *F*, *t*, *r*) with confidence intervals, effect sizes, degrees of freedom and *P* value noted<br>*Give P values as exact values whenever suitable.*
☒	☐	For Bayesian analysis, information on the choice of priors and Markov chain Monte Carlo settings
☒	☐	For hierarchical and complex designs, identification of the appropriate level for tests and full reporting of outcomes
☒	☐	Estimates of effect sizes (e.g. Cohen's *d*, Pearson's *r*), indicating how they were calculated

Our web collection on statistics for biologists contains articles on many of the points above.

Software and code

Policy information about availability of computer code

Data collection	*Provide a description of all commercial, open source and custom code used to collect the data in this study, specifying the version used OR state that no software was used.*
Data analysis	HKL2000 v7.2.0, PHASER v2.8.3, PHENIX v1.19.2, and COOT v0.9.5 are used in X-ray macrocrystallographic data processing and structure determination. PyMol v2.5, PISA v2.1.2, PyIR v1.4 were used for structure and antibody sequence analysis. Prism v9.3.1, seaborn v0.11.2 were used for binding and neutralization data analysis and visualization.

For manuscripts utilizing custom algorithms or software that are central to the research but not yet described in published literature, software must be made available to editors and reviewers. We strongly encourage code deposition in a community repository (e.g. GitHub). See the Nature Portfolio guidelines for submitting code & software for further information.

Data

Policy information about availability of data

All manuscripts must include a data availability statement. This statement should provide the following information, where applicable:

- Accession codes, unique identifiers, or web links for publicly available datasets
- A description of any restrictions on data availability
- For clinical datasets or third party data, please ensure that the statement adheres to our policy

The authors declare that the main data supporting the findings of this study are available within the article and its Supplementary Information X-ray coordinates and structure factors are deposited in the RCSB Protein Data Bank under PDB ID: 7T7B for ADI-62113 in complex with SARS-CoV-2 RBD.

Field-specific reporting

Please select the one below that is the best fit for your research. If you are not sure, read the appropriate sections before making your selection.

☒ Life sciences ☐ Behavioural & social sciences ☐ Ecological, evolutionary & environmental sciences

For a reference copy of the document with all sections, see nature.com/documents/nr-reporting-summary-flat.pdf

Life sciences study design

All studies must disclose on these points even when the disclosure is negative.

Sample size	We used similar sample sizes as in previous work (e.g. Yuan et al., 2021, Science; Liu et al., 2021, Cell Host Microbe), which we had previously determined to be sufficient sample sizes for neutralization assay.
Data exclusions	No data were excluded.
Replication	At least two biological replicates were performed for neutralization assays with SARS-CoV-2, SARS-CoV-2 variants of concern, and SARS-CoV.
Randomization	Not applicable in this study.
Blinding	Not applicable in this study.

Reporting for specific materials, systems and methods

We require information from authors about some types of materials, experimental systems and methods used in many studies. Here, indicate whether each material, system or method listed is relevant to your study. If you are not sure if a list item applies to your research, read the appropriate section before selecting a response.

Materials & experimental systems

n/a	Involved in the study	
☐	☒	Antibodies
☐	☒	Eukaryotic cell lines
☒	☐	Palaeontology and archaeology
☒	☐	Animals and other organisms
☒	☐	Human research participants
☒	☐	Clinical data
☒	☐	Dual use research of concern

Methods

n/a	Involved in the study	
☒	☐	ChIP-seq
☐	☒	Flow cytometry
☒	☐	MRI-based neuroimaging

Antibodies

Antibodies used	All of the SARS-CoV-2 antibodies used in this study were expressed in either Adimab or Wilson laboratories. ADI YYDRxG motif-containing monoclonal antibodies and antibodies under emergency use authorization were expressed and purified in Adimab as described in Methods section. Two representative YYDRxG antibodies, i.e. MOD8_P2_IgG_B11-P1369 and PZF12_P2_IgG_F7-P1369, were expressed and purified in Wilson lab as described in Methods section.<br>Antibodies used in FACS are APC-conjugated mouse anti-hemagglutinin tag (HA).11 antibody (BioLegend, cat# 901524) and PE-conjugated goat anti-human IgG polyclonal antibodies (Southern Biotech, cat# 2040-09).
Validation	All of the SARS-CoV-2 antibodies used in this study have been validated in both ELISA, SPR or BLI binding assay, and tested in pseudovirus neutralisation as reported in this study.<br>Antibodies used in FACS have been validated by their manufacture as noted in the product manual.

Eukaryotic cell lines

Policy information about cell lines

Cell line source(s)	ExpiCHO cells (Thermo Fisher Scientific Cat# A29127, RRID: CVCL_5J31) were cultured in ExpiCHO expression medium according to the manufacturer's instructions and were used to express antibody IgGs and Fabs used in this study. DH10Bac competent cells (Thermo Fisher Scientific Cat# 10361012) were cultured in LB medium and used to generate bacmids containing the SARSr RBD coding sequences. Sf9 (Cat# CRL-1711, RRID: CVCL_0549) and High five cells (Thermo Fisher Scientific Cat# B85502, RRID: CVCL_C190) were cultured in Insect-XPRESS protein-free insect cell medium (Lonza Bioscience Cat# 12-730Q) according to the manufacturer's instructions and used for generating baculoviruses and for expression of SARSr RBDs for crystallization and binding assays. HEK293T cells (ATCC Cat# CRL-3216, RRID:CVCL_0063) were cultured in DMEM (Thermo Fisher Scientific, Cat# 11960044) supplemented with 10% fetal bovine serum (Omega Scientific, Cat# FB-02),

	100U/mL Penicillin/Streptomycin (Corning, Cat# 30-002-CI), and 2mM L-glutamine (Corning, Cat# 25-005-CI) for generating SARS-CoV and SARS-CoV-2 and VOC pseudoviruses for the neutralization assay.
Authentication	None of the cell lines used were authenticated by us.
Mycoplasma contamination	Cell lines were tested mycoplasma negative.
Commonly misidentified lines (See ICLAC register)	No commonly misidentified cell lines were used in this study.

Flow Cytometry

Plots

Confirm that:

- [x] The axis labels state the marker and fluorochrome used (e.g. CD4-FITC).
- [x] The axis scales are clearly visible. Include numbers along axes only for bottom left plot of group (a 'group' is an analysis of identical markers).
- [x] All plots are contour plots with outliers or pseudocolor plots.
- [x] A numerical value for number of cells or percentage (with statistics) is provided.

Methodology

Sample preparation	EBY100 yeast were transformed with a plasmid encoding the yeast mating protein Aga2p linked to sarbecovirus RBD on the C terminus. To induce RBD expression, 0.5 OD600/ml of yeast were transferred to SGCAA media and cultured at 20C for 16-20 hours with 180 rpm shaking. Next, IgGs and hACE2 were titrated via 3-fold serial dilutions from 100 nM to 0.5 pM. RBD-expressing cells were aliquoted into 96-well plates and incubated with 100 µl of 100 nM IgG for 30 minutes on ice. Next, cells were washed twice with PBSF (1X PBS, 0.1% BSA) before secondary detection with 1:100 dilutions of APC-conjugated mouse anti-hemagglutinin tag (HA).11 antibody (BioLegend, 901524), PE-conjugated goat anti-human IgG polyclonal antibodies (Southern Biotech, 2040-09), and propidium iodide (Invitrogen, P1304MP) for 20 minutes on ice. Cells were washed twice with PBSF before analyzing via flow cytometry
Instrument	BD FACS Canto II (BD Biosciences)
Software	FlowJo v10.8.1
Cell population abundance	10,000 live (PI-low) yeast cells were collected at each antibody concentration tested to determine the mean fluorescence intensity for IgG binding (PE channel), as detected using an PE-conjugated anti-human IgG reagent. Yeast cells, as determined by FSC/SSC, represented 97% of the total population, of which ~85-95% of cells were live.
Gating strategy	All cells within the bounds of FSC/SSC were considered yeast cells. Live cells were determined as PI-low (PerCP-Cy5.5 MFI< 2000). Live yeast cells were used to obtain IgG binding signals to surface expressed RBD.

- [x] Tick this box to confirm that a figure exemplifying the gating strategy is provided in the Supplementary Information.
